# Exploring the Influence of Carbonaceous Material on the Photocatalytic Performance of the Composites Containing Bi–BiOBr and P25 TiO_2_ for NO_x_ Remediation

**DOI:** 10.1002/cphc.202500237

**Published:** 2025-06-19

**Authors:** Paransa Alimard, Stanley Cazaly, Ioanna Itskou, Hanieh Akbari, Srinivas Gadipelli, Nazila Kamaly, Flurin Eisner, Andreas Kafizas

**Affiliations:** ^1^ Department of Chemistry Molecular Science Research Hub Imperial College London 82 Wood Lane, White City Campus London W12 0BZ UK; ^2^ Science and Solutions for a Changing Planet DTP Grantham Institute for Climate Change and the Environment Imperial College London Exhibition Road, South Kensington Campus London SW7 2AZ UK; ^3^ London Centre for Nanotechnology Imperial College London South Kensington Campus London SW7 2AZ UK; ^4^ School of Engineering and Materials Science Queen Mary University of London Mile End Road London E1 4NS UK; ^5^ Department of Chemical Engineering Imperial College London South Kensington Campus London SW7 2AZ UK.; ^6^ Department of Physics & Astronomy University College London London WC1E 6BT UK; ^7^ Department of Chemical Engineering University College London London WC1E 6BT UK

**Keywords:** bismuth oxybromides (BiOBr), carbonaceous composites, nitrogen oxide (NO_x_) removals, photocatalyses, titanium dioxides (TiO_2_)

## Abstract

The Bi–BiOBr–P25 TiO_2_ composite material exhibits high and synergistic improvements in the photocatalytic activity for nitrogen oxides (NO_x _ = NO + NO_2_) removal. Herein, the influence of adding carbonaceous material to this composite, namely graphene (G), graphene oxide (GO), carbon nanotubes (CNT), and buckminsterfullerene (F) is explored; all at 1 wt%. Samples are synthesised using a one‐pot solvothermal method. The structural and morphological properties, composition, and photocatalytic performance of all samples are examined using scanning electron microscopy, carbon–hydrogen–nitrogen elemental analysis, high‐resolution transmission electron microscopy, X‐ray diffraction, Raman spectroscopy, attenuated total reflectance–Fourier transform infrared spectroscopy, ultraviolet–visible (UV–vis) spectroscopy, X‐ray photoelectron spectroscopy, N_2_ sorption at 77 K, photoluminescence spectroscopy, diffuse reflectance transient absorption spectroscopy), and photocatalytic testing against NO_x_ gas in accordance with ISO protocol (22197‐1:2016). Among the studied carbonaceous composites, the composite including GO shows the highest performance toward NO_x_ remediation. For reactions in NO gas, it shows a combined higher NO_x_ removal rate (21.9%) than its parent materials P25 (8.7%), Bi–BiOBr (6.5%), and GO (0%). For reactions in NO_2_ gas, it shows a higher NO_x_ removal rate (≈15%) than its parent materials P25 (≈10%), Bi–BiOBr (≈5%), and GO (0%).

## Graphical abstract

Bi/BiOBr/P25 TiO_2_ composites are synthesized via a solvothermal method incorporating 1 wt% of a carbon‐based material (graphene oxide [GO], graphene, carbon nanotubes, or buckminsterfullerene). The GO–based composite exhibits the highest photocatalytic NO_x_ removal efficiency (≈22% NO and ≈15% NO_2_) with these synergetic improvements attributed to increased charge separation, surface area, and light absorption.

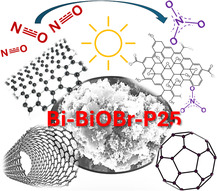

## 1 Introduction

Nitrogen oxides (NO_x_), including nitric oxide (NO) and nitrogen dioxide (NO_2_), form during the fossil fuel combustion.^[^ ^1^, ^2^ ^]^ These pollutants cause acid rain, harm ecosystems, and aquatic life, and contribute to haze and ground‐level ozone, leading to millions of premature deaths worldwide.^[^ ^1^, ^3^ ^]^ Therefore, reducing the atmospheric concentration of NO_x_ pollution is a pressing issue. It has been well‐documented that titanium dioxide (TiO_2_) is capable of remediating NO_x_ pollution under the action of photocatalysis, and has been implemented in several commercial building materials, including cement, tiles, paints, and spray‐coatings.^[^ ^4^ ^]^ However, the wide bandgap (3.2 eV) of TiO_2_ limits its activity only to the UV portion of the electromagnetic spectrum.^[^ ^3^ ^]^ To overcome this limitation, TiO_2_ has been coupled to narrower bandgap semiconductors, extending absorption into the visible while also prolonging the lifespan of photogenerated charge carriers.

Bismuth oxybromide (BiOBr) is a promising photocatalyst for NO_x_ remediation that possesses a narrower bandgap (≈2.8 eV) than TiO_2_ and is also non‐toxic and easy to manufacture.^[^ ^5^, ^6^, ^7^ ^]^ Recent studies have demonstrated that forming a composite of BiOBr and TiO_2_ can significantly enhance photocatalytic activity for pollution remediation.^[^ ^8^, ^9^, ^10^ ^]^ Moreover, the incorporation of Bi nanoparticles, into such a composite, can further enhance activity, through the enhanced spatial separation of charge and potential co‐catalytic and plasmonic effects.^[^ ^11^, ^12^, ^13^ ^]^

Carbon‐based nanomaterials, including graphene (G), reduced graphene oxide (rGO), buckminsterfullerene (F), and carbon nanotubes (CNTs), exhibit remarkable surface functionality with electrical conductivity^[^ ^14^, ^15^, ^16^ ^]^ and possess a tunable bandgap,^[^ ^17^, ^18^, ^19^, ^20^, ^21^ ^]^ enabling them to function as either semiconductors or quasi‐metals.^[^ ^22^, ^23^, ^24^, ^25^ ^]^ When incorporated with semiconductors to form composites, these materials extend light absorption into the visible region and can prolong the lifespan of charge carriers.^[^ ^26^, ^27^ ^]^ Such carbon‐based materials have been shown to possess significant capacity for the adsorption of NO and NO_2_ gas.^[^ ^28^, ^29^, ^30^ ^]^

Graphene‐related materials exhibit remarkable physicochemical properties, including exceptional electron mobility, which is attributed to its unique 2D structure and *sp* ^2^‐hybridized carbon framework. Graphene's electronic band structure results in a zero‐gap material.^[^ ^31^ ^]^ In graphene, electrons act as relativistic Dirac fermions, possessing an extremely low effective mass, which allows them to traverse the lattice with minimal scattering.^[^ ^32^ ^]^ Additionally, graphene has a large surface area,^[^ ^33^ ^]^ exceptional thermal conductivity,^[^ ^34^ ^]^ and impressive optical transparency.^[^ ^35^ ^]^ Graphene oxide (GO) consists of a carbon framework decorated with a combination of epoxy, hydroxyl, carbonyl, and carboxyl functional groups.^[^ ^36^ ^]^ GO offers bandgap tuning and facilitates the exploration of graphene's dynamic optical properties across various spectral ranges.^[^ ^36^ ^]^ Buckminsterfullerene consists of a 12 pentagon and 20 hexagon structure,^[^ ^37^ ^]^ and shows remarkable features including high electrochemical stability within a compact structure.^[^ ^38^ ^]^ The nanometer‐scale size and unique morphology of buckminsterfullerene endow it with physical and chemical properties distinct from conventional carbon materials.^[^ ^39^ ^]^ CNTs, which can be considered 1D cylindrical structures formed by rolling graphene sheets, exhibit varying conductivity depending on how the hexagonal graphene lattice is rolled and the radius of the resulting cylindrical structure.^[^ ^40^ ^]^ The hexagonal rings of carbon can form zigzag (semiconducting), armchair (quasi‐metallic), and chiral (semiconducting) structures.^[^ ^40^ ^]^ The insertion of non‐hexagonal defects causes bent (kink), branched, and coiled structures.^[^ ^40^ ^]^ Also, CNTs exhibit diverse physicochemical properties depending on their length, nanotube diameter, chirality, and the number of layers. This structural variety results in different band structures, as well as both quasi‐metallic and semiconducting properties.

Trapalis et al. studied the NO_x_ removal performance of TiO_2_/G and TiO_2_/rGO, reporting that both G and rGO in the composite enhance photocatalytic efficiency for NO_x_ oxidation, especially under visible light; increasing total NO_x_ removal and reducing NO_2_ emissions.^[^ ^41^ ^]^ The group reported that TiO_2_/rGO exhibited better performance than TiO_2_/G, with the optimal GO loading being 0.1 wt%. In another study, Pham et al. reported that CNT in TiO_2_/CNT composites increase the photooxidation of NO_x_.^[^ ^42^ ^]^ They noted that the TiO_2_/CNT composite with 0.7 wt% CNT exhibits the highest photocatalytic performance. Qi et al. used density functional theory (DFT) calculations to investigate the electronic structure of the TiO_2_/C_60_ composite.^[^ ^43^ ^]^ They found that the photocatalytic activity of anatase TiO_2_ improves with fullerene modification, with an optimal loading of 2.0 wt%. They reported that incorporating C_60_ on the TiO_2_ surface narrows its bandgap and introduces doping states between the valence and conduction bands.

Herein, we study the potential benefit of incorporating carbonaceous materials into Bi–BiOBr–P25 TiO_2_ composites for photocatalytic NO_x_ remediation. More specifically, 0D buckminsterfullerene, 1D multi‐walled CNTs (MWCNTs), and 2D GO and rGO are explored for this purpose. Their impact on the structure, morphology, and charge carrier behavior, along with photocatalytic activity and selectivity of NO and NO_2_ remediation, is investigated under UVA lighting conditions.

## 2 Experimental Section

### 2.1 Chemicals and Materials

Potassium bromide (99%), bismuth (III) nitrate (98%), graphite powder (<20 μm), sodium borohydride (99%), nitric acid (70%), potassium chlorate (≥99.0%), and ethylene glycol (99%) were obtained from Sigma‐Aldrich. TiO_2_ (P25) was acquired from Thermo Scientific. NO and NO_2_ gases (100 ppm concentration diluted in N_2_) were purchased from BOC UK. Ethanol absolute (99.5%), sulfuric acid (98%), and hydrochloric acid (37%) were purchased from VWR. Cetyltrimethylammonium bromide (CTAB) (98%), MWCNT of 10–20 nm (diameter) and 5–15 μm (length), and buckminsterfullerene C_60_ (99.5%) were purchased from Tokyo Chemical Industry (TCI).

#### 2.1.1 GO Synthesis

GO was synthesized using a modified Staudenmaier method.^[^ ^44^, ^45^, ^46^, ^47^ ^]^ The process entailed precise temperature control achieved by combining 9 mL concentrated nitric acid and 18 mL sulfuric acid in a 1:2 vol% ratio within an ice bath. Subsequently, 1.0 g of graphite powder was introduced into the acid mixture under vigorous stirring, ensuring uniform dispersion and preventing agglomeration. To mitigate abrupt temperature increases and explosive chlorine dioxide gas formation, a gradual addition of 11 g of potassium chlorate was added over the course of one hour, while stirring and maintaining the reaction flask within the ice bath. After the complete dissolution of potassium chlorate, the mixture was continuously and vigorously stirred for 96 h at room temperature. Upon completion of the reaction, the mixture was carefully poured into 1 L of deionized (DI) water, followed by a decantation process. To eliminate sulphate ions, the GO underwent a process of redispersion in 5 vol% HCl solutions, followed by multiple rounds of centrifugation and redispersion in DI water. Subsequently, the graphite oxide slurry was dried in an oven at 60 °C for 48 h.

#### 2.1.2 Graphene (G) Synthesis

The reduction of the GO, synthesized in the previous step, was carried out following the method described by Guex et al.^[^ ^48^ ^]^ First, 70 mg of GO was dispersed in 35 mL of DI water using an ultrasonic bath (VWR ultrasonic cleaner, 30 W, 45 kHz). Sodium borohydride (397 mg, 0.01 mole) was then added to the mixture to prepare a 0.3 M solution. The vial was heated to 80 °C and stirred for 195 min. After the reaction, the samples were cooled down in an ice bath. The sample was then isolated by centrifugation at 8500 rpm for 30 min, with the supernatant replaced with fresh DI water. This process was repeated three times to remove any residual ions. Then the washed sample was freeze‐dried at 0.047 mbar and −55.8 °C (Telstar CRYODOS freeze dryer).

#### 2.1.3 Bi/BiOBr Synthesis

The Bi–BiOBr synthesis method followed a procedure outlined in our previous work.^[^ ^13^ ^]^ First, 3 mmol (1.46 g) of bismuth (III) nitrate was added to 17 mL of ethylene glycol and dissolved under ultrasonication. Then, 3 mmol (1.09 g) of CTAB was added to 18 mL of ethylene glycol and sonicated using the ultrasonic bath until dissolved. The bismuth solution was added to the CTAB solution dropwise (one drop per second), and then the final solution was magnetically stirred for 30 min. The resulting suspension was then poured into a 200 mL Teflon‐lined autoclave, where it underwent solvothermal treatment at 180 °C for 12 h.^[^ ^49^ ^]^ The synthesized brown precipitate was isolated by centrifugation (7500 rpm), washed thoroughly with water and ethanol three times, and finally dried at 60 °C overnight.

#### 2.1.4 Bi–BiOBr–P–GO1 Composite Synthesis

Bi–BiOBr composites with P25 TiO_2_ and GO were produced with a Bi to Ti ratio of 4.4 at% and GO of 1 wt%. The synthesis method was adapted to our previous study,^[^ ^13^ ^]^ with the precise amounts of the materials used as listed in **Table** **1**. Initially, bismuth (III) nitrate was added to 17 mL of ethylene glycol and subjected to bath sonication until complete dissolution. Subsequently, CTAB was dissolved in 18 mL of ethylene glycol and sonicated using an ultrasonic bath until it dissolved. In another breaker, P25 TiO_2_ was dispersed in a 35 mL aqueous solution of GO and sonicated for 30 min. Then, the mixture containing GO and P25 was added to Bi solution dropwise (one drop per second), followed by the addition of the CTAB solution dropwise to the former solution (one drop per second). Then, the mixture was sonicated using an ultrasonic horn probe (SONICS Vibra‐Cell) for 5 min (amplitude 30%, 3 s on 3 s off). The mixture was transferred to a 200 mL Teflon‐lined autoclave and solvothermally treated at 180 °C for 12 h. Following the treatment, the resulting precipitate was separated using filter paper under gravity, washed with water and ethanol three times, and subsequently freeze‐dried at 0.047 mbar and −55.8 °C (Telstar CRYODOS freeze dryer). The composite was denoted Bi–BiOBr–P–GO1.

**Table 1:** The amounts of chemicals used to prepare Bi–BiOBr–P–G1O1, Bi–BiOBr–P–G1, Bi–BiOBr–P–CNT1, and Bi–BiOBr–P–F1 composites.

	Bi–BiOBr–P–GO1	Bi–BiOBr–P–G1	Bi–BiOBr–P–CNT1	Bi–BiOBr–P–F1
Bi(NO_3_)_3_·5H_2_O	3.0 mmol (1.46 g)	3.0 mmol (1.46 g)	3.0 mmol (1.46 g)	3.0 mmol (1.46 g)
CTAB	3.0 mmol (1.09 g)	3.0 mmol (1.09 g)	3.0 mmol (1.09 g)	3.0 mmol (1.09 g)
P25	13.13 mmol (1.05 g)	13.13 mmol (1.05 g)	13.13 mmol (1.05 g)	13.13 mmol (1.05 g)
GO/G/CNT/F	20 mg	20 mg	20 mg	20 mg

#### 2.1.5 Bi–BiOBr–P–C (C: Graphene [G], CNT, and Fullerene [F]) Composite Synthesis

Bi–BiOBr–P–C composites (where C represents G, CNT, or F) were synthesized following the same procedure as Bi–BiOBr–P–GO1, but the carbon‐based material was dispersed in CTAB solution rather than in the mixture of P25 TiO_2_ and water before being added to the Bi solution. The reason being that CTAB, as a cationic surfactant, can help disperse hydrophobic carbonaceous materials. The exact quantities of the materials used are provided in Table 1, with all samples produced in this study listed in **Table** **2** and Figure S1, Supporting Information.

**Table 2:** Nomenclature of all synthesized samples, including synthesis conditions.

Sample	Synthetic method	Synthesis duration	Atomic ratio Ti/Bi
GO	Staudenmaier	98 h	–
G	Chemical reduction	4 h	–
Bi–BiOBr	Solvothermal	12 h	–
Bi–BiOBr–P–GO1	Solvothermal	12 h	0.2
Bi–BiOBr–P–G1	Solvothermal	12 h	2.2
Bi–BiOBr–P–CNT1	Solvothermal	12 h	4.4
Bi–BiOBr–P–F1	Solvothermal	12 h	6.6

### 2.2 Physical Characterization

High‐resolution scanning electron microscopy (SEM) was conducted using a Zeiss Sigma 300 field emission gun SEM system. Conductive carbon adhesive tape was used for SEM images to fix the samples on SEM mountings. A Flash 2000 Organic Elemental Analyzer (Thermo Scientific), carbon–hydrogen–nitrogen (CHN) elemental analyzer, was used to determine the carbon, hydrogen, and nitrogen composition in samples. Transmission electron microscopy (TEM) and scanning transmission electron microscopy were conducted using a JOEL JEM‐2100Plus electron microscope and a high‐resolution JOEL JEM‐2100 F field‐emission electron microscope at 200 KV. A fast Fourier transform was employed to obtain digital‐selected‐area electron diffraction patterns from the high‐resolution TEM (HR‐TEM), adopting ImageJ software. X‐ray diffraction (XRD) patterns were obtained with a Bruker D2 Phaser diffractometer, featuring parallel beam optics and a Lynx‐Eye detector. Using a Cu source (V = 30 kV, I = 10 mA) with Cu K_α1_ radiation (*λ* = 1.54056 Å) and Cu K_a2_ radiation (*λ* = 1.54439 Å), X‐rays were generated and emitted with an intensity ratio of 2:1. Patterns were measured between 5° ≤ 2Θ ≤ 80° for samples with a step size of 0.02°. The patterns were analyzed and compared with reference standards from the Inorganic Crystal Structure Database (ICSD). Raman spectroscopy was carried out at room temperature using a Bruker Senterra II Raman microscope with a 532 nm laser excitation wavelength, within 50–1000 cm^−1^ (resolution: 1.5 cm^−1^). Attenuated total reflection–Fourier transform infrared (ATR–FTIR) was measured using an Agilent Cary 630 analyzer from 400 to 4000 cm^−1^ (resolution: 0.2 cm^−1^). UV–vis diffuse reflectance spectroscopy (DRS) measurements from 200 to 800 nm (resolution: 1 nm) were measured using a Shimadzu UV‐2600 spectrophotometer with an integrating sphere, employing a barium sulphate powder‐pressed disc as a diffuse reflectance standard. UV–visible DRS was performed from 200 to 800 nm. The diffuse reflectance data was converted into the Kubelka–Munk function (F(R)), which is directly proportional to absorbance based on the Kubelka–Munk theory (Equation (1)):

(1) $$F\left(R\right)=\frac{K}{S}=\frac{{\left(1-R\right)}^{2}}{2R}$$

where *R* is the diffuse reflectance, and *K* and *S* represent the absorption and scattering coefficients, respectively. By using the Tauc plot method (Equation (2)), we derived the indirect (*n* = 0.5) allowed bandgap energy values.^[^ ^50^, ^51^, ^52^, ^53^, ^54^, ^55^ ^]^ *F*(*R*) serves as a proxy for the absorption coefficient, denoted by α:

(2) $${\left(F\left(R\right).hv\right)}^{n}$$

where *h* and *v* are Planck's constant and the frequency of light, respectively. These variables were plotted against energy, and by extrapolating to the energy axis, the bandgap energies were determined.

X‐ray photoelectron spectroscopy (XPS) measurements were conducted using a Thermo Scientific K‐α^+^ surface analysis X‐ray photoelectron spectrophotometer with a MXR3 Al K_α_ monochromatic X‐ray source. The analyzer's vacuum level was maintained at 8 × 10^−^^8^ mbar during the test, covering the survey scan as well as the C1s, O1s, Ti2p, Bi4f, and Br3d core levels. N_2_‐sorption isotherms were obtained at −196 °C using a Micrometrics 3Flex volumetric sorption analyzer. Before the measurements, the samples were degassed ex situ using a Micrometrics VacPrep Degasser at 120 °C overnight at 2 × 10^−^^5^ bar, followed by in situ degas using the 3Flex sorption analyzer at 110°C for 4 h at 7 × 10^−^^5^ bar. The specific surface areas of the samples were determined using the Brunauer–Emmett–Teller (BET) method.^[^ ^56^ ^]^ The total pore volume (*V*_tot_) was estimated from the amount of adsorbed N_2_ at P/P_0 _= 0.99. The micropore volume (*V*_micro_) was calculated using the Dubinin–Radushkevich method,^[^ ^57^ ^]^ and the mesopore volume (*V*_meso_) was obtained by subtracting *V*_micro_ from *V*_total_. Photoluminescence (PL) spectroscopy was carried out on samples using an Edinburgh Instruments FLS 1000 PL spectrometer. The m PL signals over the 350–800 nm range for all the samples were seen when the detector used a 5 nm bandwidth slit and a 395 nm long pass filter was placed before the detector and samples were excited with 375 nm light.

### 2.3 Transient Absorption Spectroscopy

Transient absorption spectroscopy (TAS) was performed in diffuse reflection mode over a timescale ranging from microseconds to seconds, utilizing a neodymium‐doped yttrium aluminum garnet laser (OPOTEK Opolette 355 II, ≈6 ns pulse width) as the excitation source, emitting UV light at 355 nm from the third harmonic (≈220 μJ cm^−^^2^ per pulse, repetition rate of 0.67 Hz). A 100 W Bentham IL1 quartz halogen lamp served as the source for generating probe light. Long‐pass filters from Comar Instruments were positioned between the probe lamp and the sample to reduce short‐wavelength irradiation. Transient changes in the sample's diffuse reflectance were captured using a 2‐inch diameter lens with a 2‐inch focal length and directed into a monochromator (Oriel Cornerstone 130). Measurements were taken at wavelengths between 600 and 1100 nm, in 100 nm intervals. Time‐resolved changes in diffuse reflectance were detected using a Si photodiode (Hamamatsu S3071). Data for times faster than 3.6 ms were recorded with an oscilloscope (Tektronix DPO3012) via an amplifier box (Costronics), while data for times slower than 3.6 ms were recorded using a National Instrument DAQ card (NI USB‐6251). Each kinetic trace was averaged over at least 100 laser pulses and measurements. Experiments were triggered by a photodiode (Thorlabs DET10A) that responded to scattered laser light. Data acquisition and processing were handled using custom‐developed LabVIEW software. For each experiment, a few milligrams of the sample were placed between two microscope slides secured with masking tape, and measurements were carried out in air. In the TAS study, GO, G, CNT, F, and Bi–BiOBr–P–C (C: G, GO, CNT, and F) composites, as well as Bi–BiOBr and P25 as reference materials, were tested.

### 2.4 Photocatalytic Activity Measurement

The photocatalytic NO removal experiments on the synthesized samples were carried out using a continuous flow reactor within an air pollution simulation chamber designed by the ISO 22197‐1:2016 standard. For each sample, 50 mg of the photocatalyst was dispersed in 3 mL of ethanol and ultrasonicated for 10 min. The resulting suspension was uniformly applied to a 5 × 10 cm float glass substrate using an airbrush gun (Fengda Professional‐130, 0.3 mm nozzle) and left for 15 min to dry at room temperature until the ethanol had fully evaporated.

The sample was placed at the center of the reactor. The experiments were performed under the standard conditions specified by ISO; 50% relative humidity (RH) at room temperature with a flow rate of 3000 sccm of NO/NO_2_ gas diluted with air to achieve a concentration of 1 ppm. Changes in gas‐phase NO and NO_2_ concentrations were measured each minute using an Ecotech Serinus 40 chemiluminescence NO_x_ analyzer. Each experiment consisted of 15 min of dark time, 60 min of irradiation (2 × 15 W Sankyo FL15T8BLB, 300–400 nm), followed by 15 min of dark time. A ThorLabs PM100D power energy meter with an S120VC sensor was used to measure the power output of the light source to achieve ≈1.00 mW cm^−2^. A Fischer Haar hygrometer was used to measure the RH.

When the target gas was NO, the following equations were used to determine changes. The percentage of NO removal was measured using Equation (3):

(3) $$\text{NO removal }\left(\text{wt \%}\right)=\frac{{\left[\text{NO}\right]}_{i}-{\left[\text{NO}\right]}_{f}}{{\left[\text{NO}\right]}_{i}}\times 100$$

The NO_x_ removal percentage was measured using Equation (4):

(4) $${\text{NO}}_{x}\text{ removal }\left(\text{wt \%}\right)=\frac{{\left[\text{NO}\right]}_{i}\;-{\left[{\text{NO}}_{x}\right]}_{f}}{{\left[\text{NO}\right]}_{i}\;}\times 100$$

The NO_2_ generation percentage was measured using Equation (5):

(5) $${\text{NO}}_{2}\text{ generation }\left(\text{wt \%}\right)=\frac{{\left[{\text{NO}}_{2}\;\right]}_{f}\;-{\left[{\text{NO}}_{2}\;\right]}_{i}}{{\left[\text{NO}\right]}_{i}}\times 100$$

And NO_2_ selectivity% was measured using Equation (6):

(6) $${\text{NO}}_{2}\text{ selectivity}=\left(\frac{{\left[{\text{NO}}_{\text{2}}\right]}_{\text{out}}-{\left[{\text{NO}}_{\text{2}}\right]}_{\text{in}}}{{\left[\text{NO}\right]}_{\text{in}}-{\left[\text{NO}\right]}_{\text{out}}}\right)\times \;100$$

where []_*i*_ and []_*f*_ are the average concentrations of each gas in the dark time stream, initial concentration, and light time stream, final concentration, respectively.

When the NO_x_ test was conducted under NO_2_ gas, the NO_2_ removal percentage was measured using Equation (7):

(7) $${\text{NO}}_{2}\text{ removal }\left(\text{wt \%}\right)=\frac{{\left[{\text{NO}}_{2}\;\right]}_{i}\;-{\left[{\text{NO}}_{2}\;\right]}_{f}}{{\left[{\text{NO}}_{2}\;\right]}_{i}}\times 100$$

The NO_x_ removal percentage was measured using Equation (8):

(8) $${\text{NO}}_{x}\text{ removal }\left(\text{wt \%}\right)=\frac{{\left[\text{NO}\right]}_{i}\;-{\left[{\text{NO}}_{x}\right]}_{f}}{{\left[\text{NO}\right]}_{i}\;}\times 100$$

The NO generation percentage was measured using Equation (9):

(9) $$\text{NO generation }\left(\text{wt \%}\right)=\frac{{\left[\text{NO}\right]}_{f}-{\left[\text{NO}\right]}_{i}}{{\left[{\text{NO}}_{2}\right]}_{i}}\times 100$$

And the NO selectivity% was measured using Equation (10):

(10) $$\text{NO selectivity}=\left(\frac{{\left[\text{NO}\right]}_{\text{out}}-{\left[\text{NO}\right]}_{\text{in}}}{{\left[{\text{NO}}_{2}\right]}_{\text{in}}-{\left[{\text{NO}}_{2}\right]}_{\text{out}}}\right)\times \;100$$

## 3 Results and Discussion

### 3.1 Overview of the Samples Produced

The focus of this study is on the interaction between carbon‐based materials and Bi–BiOBr–P composites and their impact on photocatalytic NO_x_ remediation. A one‐pot solvothermal method was used to synthesize a range of 1 wt% carbon‐based composites, with graphene (G), GO, CNT, and buckminsterfullerene (F). Except for GO, which is hydrophilic, the other carbon‐based materials were dispersed in ethylene glycol with the assistance of CTAB. CTAB serves both as a source of Br to form BiOBr and as a cationic surfactant, helping the hydrophobic carbon‐based materials disperse more easily by reducing the surface tension. In our previous study,^[^ ^13^ ^]^ the optimal Ti/Bi atomic ratio was found to be 4.4, where this ratio was maintained in the current study. Photographs of the samples produced and studied herein are shown in Figure S1, Supporting Information. The commercial P25 TiO_2_ powder was a brilliant white, Bi–BiOBr was a brown powder, Bi–BiOBr–P–GO1, Bi–BiOBr–P–G1, and Bi–BiOBr–P–CNT1 were grey–blue powders, Bi–BiOBr–P–F1 was a white–grey powder. The mixture of Bi–BiOBr and P25, combined in the same ratio as in Bi–BiOBr–P, appeared light brown in color, and Bi–BiOBr–P was white–blue. The color difference between the Bi–BiOBr and P25 mixture (light brown) and the Bi–BiOBr–P composite (white–blue) suggests a structural change, confirming the successful formation of the Bi–BiOBr–P material.

### 3.2 SEM Imaging and CHN Analysis

**Figure 1:**
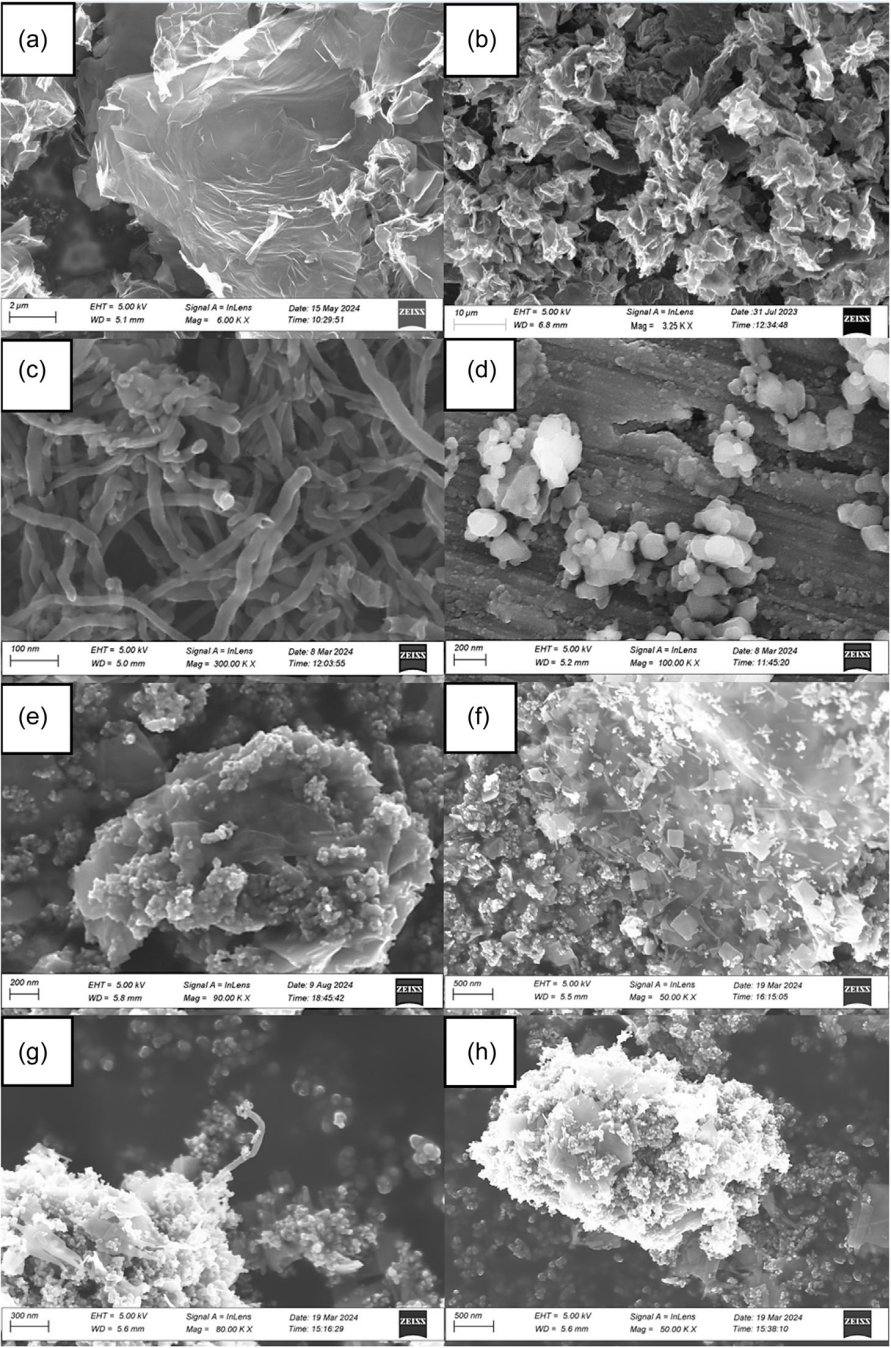
SEM images of a) GO, b) G, c) CNT, d) F, e) Bi–BiOBr–P–GO1, f) Bi–BiOBr–P–G1, g) Bi–BiOBr–P–CNT1, and h) Bi–BiOBr–P–F1 samples.

To investigate the morphology of the samples SEM imaging was employed (**Figure** **1**). The size and morphology of GO, G, CNT, and F are shown in Figure 1a–d, respectively. Analysis of GO reveals how graphite was exfoliated, resulting in a broad size distribution of the sheets in the micron size range (Figure 1b). However, the G sheets appear shrunken compared to the GO sheets. The CNTs showed diameters ≈10 nm wide and lengths in the region of 200–300 nm, and F appeared to agglomerate into clumps ≈200 nm in diameter. Figure 1e–h shows images of the composites formed between these carbon‐based materials and Bi–BiOBr–P for GO, G, CNT, and F, respectively. Figure 1e,f, shows how the Bi–BiOBr and P particles were decorated and wrapped in thin layers of GO and G sheets, respectively. Figure 1g illustrates the incorporation of CNTs into the structure of the Bi–BiOBr–P–CNT1 composite. And Figure 1h depicts an SEM image of the Bi–BiOBr–P–F1 composite, where discrete F particles were not able to be observed, likely due to their sub‐nanometer particle size in low concentrations. The SEM images of Bi–BiOBr and P25 are presented in Figure S24a,b (Supporting Information), respectively.

CHN elemental analysis results confirmed that the expected carbon loading in Bi–BiOBr–P–GO1, Bi–BiOBr–P–G1, Bi–BiOBr–P–CNT1, and Bi–BiOBr–P–F1 composites was ≈1 wt% (**Table** **3**).

**Table 3:** Elemental analysis CHN of Bi–BiOBr–P–GO1, Bi–BiOBr–P–G1, Bi–BiOBr–P–CNT1, and Bi–BiOBr–P–F1 composites.

Element	Bi–BiOBr–P–GO1	Bi–BiOBr–P–G1	Bi–BiOBr–P–CNT1	Bi–BiOBr–P–F1
Carbon [wt%]	1.51	1.1	1.47	1.34
Hydrogen [wt%]	0.04	0.10	0.01	0.02
Nitrogen [wt%]	0.00	0.06	0.00	0.01

### 3.3 HR‐TEM Imaging

**Figure 2:**
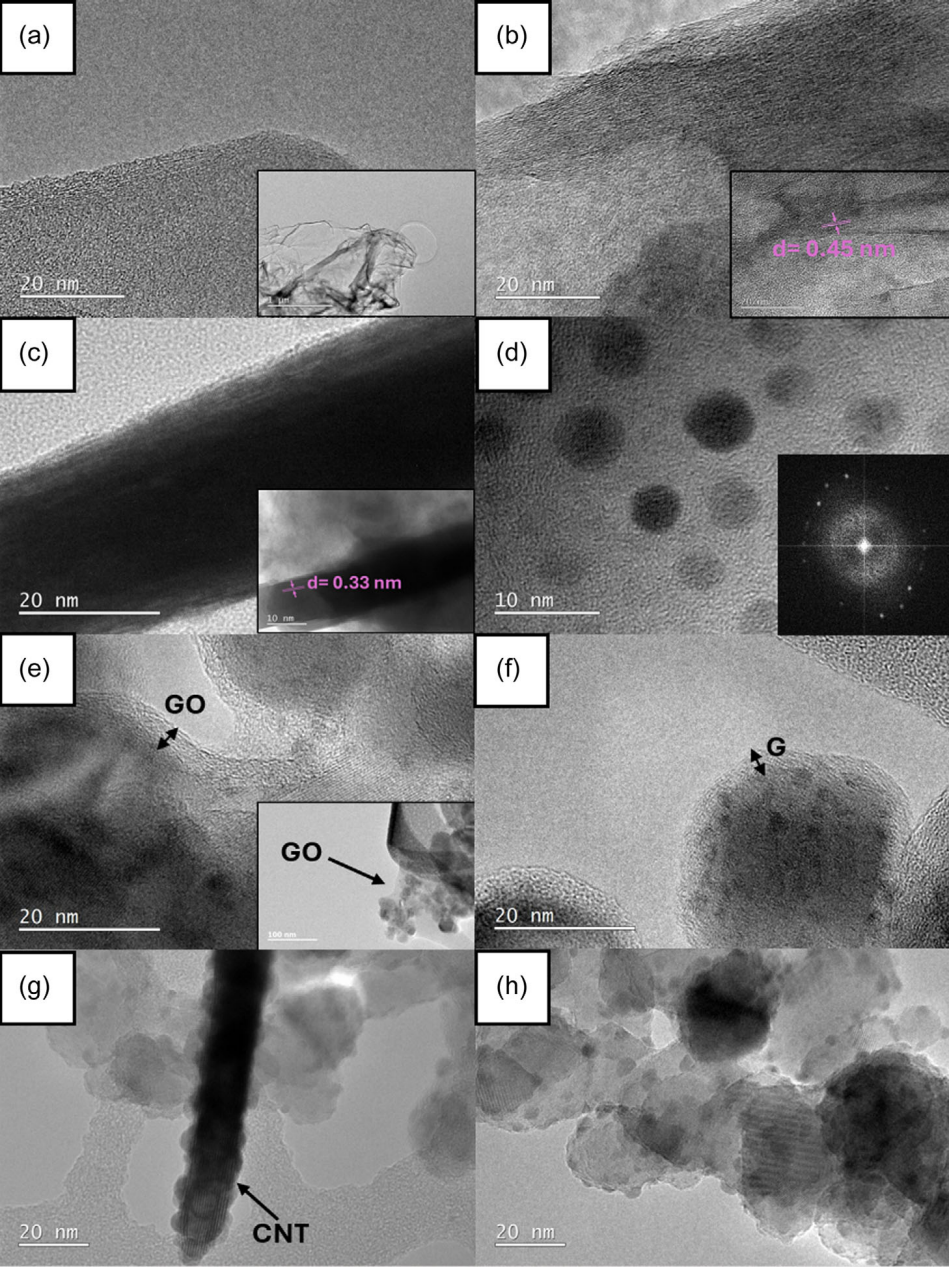
TEM images of a) GO, b) G, c) CNT, d) F, e) Bi–BiOBr–P–GO1, f) Bi–BiOBr–P–G1, g) Bi–BiOBr–P–CNT1, and h) Bi–BiOBr–P–F1 samples.

HR‐TEM images of GO, G, CNT, F, Bi–BiOBr–P–GO1, Bi–BiOBr–P–G1, Bi–BiOBr–P–CNT1, and Bi–BiOBr–P–F1 are shown in **Figure** **2**. The TEM image of GO (Figure 2a) indicates that its layers are thinner than the carbon support from the sample holder (≈28–30 nm thick). G (Figure 2b) possesses wavy sheets of carbon with a d‐spacing of 0.45 nm, corresponding to the (002) graphite crystal plane. This indicates that the reduction process of GO to form G in the monitored section was not complete, resulting in a wider d‐spacing compared to that of a graphite standard.^[^ ^58^ ^]^ CNT (Figure 2c) possesses the expected cylindrical structure of nanotubes with an external diameter of ≈30 nm and d‐spacing of 0.33 nm in the (002) crystal plane.^[^ ^59^ ^]^ In Figure 2d, the F particles show a d‐spacing of 0.84 nm, corresponding to the (111) crystal plane of buckminsterfullerene, with particle sizes ranging between 5 and 7 nm. However, in some areas of analysis, the F particles exhibited irregular shapes and significant aggregation (Figure S2, Supporting Information).^[^ ^60^ ^]^ In our previous study,^[^ ^13^ ^]^ the phases of the components in Bi–BiOBr–P were identified through TEM analysis, revealing rhombohedral Bi (*R*–3*mH*), tetragonal BiOBr (*P*4/*nmm*), and tetragonal anatase TiO_2_ (*I*4_1_/*amd*). Larger BiOBr flakes, of the micron‐scale in width and ≈10 nm in thickness, were decorated with rounded TiO_2_ particles ≈20 nm in width and spherical Bi nanoparticles ≈5 nm in diameter. Herein, we provide an analysis of the morphology of Bi–BiOBr–P–GO1, Bi–BiOBr–P–G1, Bi–BiOBr–P–CNT1, and Bi–BiOBr–P–F1 composites as observed through TEM. The Bi–BiOBr–P–GO1 composite (Figure 2e) showed Bi–BiOBr–P particles intimately covered by GO sheets that wrapped around these particles and therefore likely formed a distinct heterojunction interface.^[^ ^61^ ^]^ A similar degree of contact can be seen between Bi–BiOBr–P and G in the Bi–BiOBr–P–G1 composite (Figure 2f). In the Bi–BiOBr–*P*–CNT1 composite (Figure 2g), the CNTs are decorated with Bi–BiOBr–P with high surface coverage and in Bi–BiOBr–P–F1 (Figure 2h), the buckminsterfullerene coats the Bi–BiOBr–P particles, with round particles ≈5 nm in size. Jiang et al. examined the morphology of P25, and the corresponding TEM image can be found in their publication.^[^ ^62^ ^]^ The TEM image of Bi–BiOBr–P is shown in Figure S24c, Supporting Information.

### 3.4 XRD and Raman Spectroscopy

The XRD pattern of GO (Figure S3, Supporting Information) exhibits a strong and sharp peak at 2Θ = 12.95° corresponding to the (001) plane,^[^ ^60^ ^]^ and the other peak with relatively weak intensity appears at 2Θ = 42.17°, corresponding to the (004) plane. Apart from 2Θ = 42.4° (100), no other peaks are related to the hexagonal structure of graphite, which typically can be seen at 2Θ = 26.5° (002), 2Θ = 44.6° (101), and 2Θ = 54.7° (004) were observed in the XRD pattern of the synthesized GO.^[^ ^59^ ^]^ The interplane distance in typical graphite is 3.37 Å,^[^ ^63^ ^]^ while the reported interplanar spacing of a typical GO is about 7 Å,^[^ ^64^, ^65^ ^]^ revealing that the oxygen functional groups in GO increase the d‐spacing of the planes. As shown in Figure S3 (Supporting Information), the full‐width at half‐maximum (FWHM) of the (001) peak of GO was obtained by peak fitting adopting Lorentz peak function. The average crystal size of the prepared GO fragments was calculated to be ≈4.6 nm based on the Scherrer equation, as shown in Equation (11).^[^ ^59^ ^]^

(11) $$L_{c}=\frac{K}{\beta \;\cos\theta }$$

where *L*_*c*_ is the average crystal size, K is the dimensionless constant (0.9), *λ* is the X‐ray wavelength (0.154056 nm), *β* is the FWHM and Θ is the Bragg angle. Using Bragg's law, Equation (12), the interplane spacing can be measured:^[^ ^59^ ^]^

(12) $$d_{hkl}=\frac{n\lambda }{2\sin\theta }\;$$

where *d*_*hkl*_ is interplane distance, *n* is diffraction order for first order (1), *λ* is the X‐ray wavelength (0.154056 nm), and Θ is the reflection angle for (001) crystal facet.^[^ ^59^ ^]^ The *d*_*hkl*_ of the prepared GO using Equation (10) was measured to be ≈0.69 nm, which is in agreement with the reported d‐spacing of GO from other studies.^[^ ^66^ ^]^ The number of GO layers in the measured fragment can be obtained using the Equation (13):^[^ ^59^ ^]^

(13) $$N=\frac{L_{c}}{d_{hkl}}+1$$

where *N* is the number of layers in the fragment, and *L*_*c*_ and *d*_*hkl*_ were defined earlier. For the GO herein, the number of layers was ≈8.

**Figure 3:**
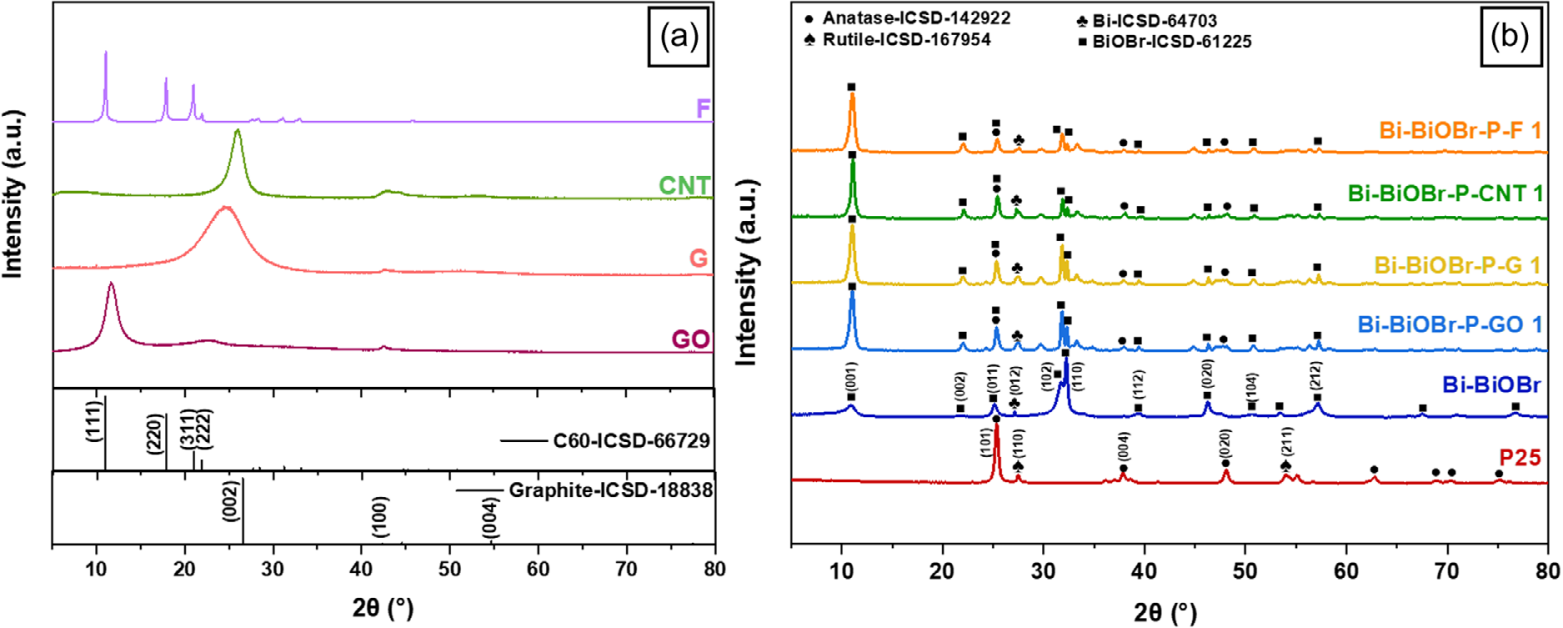
The XRD patterns of a) GO, G, CNT, and F, and b) P25, Bi–BiOBr, Bi–BiOBr–P–GO1, Bi–BiOBr–P–G1, Bi–BiOBr–P–CNT11, and Bi–BiOBr–P–F1 samples.

The XRD patterns of GO, G, CNT, and F are illustrated in **Figure** **3**a. In the XRD pattern of G, the crystallite size (*L*_*c*_) was determined to be ≈1.5 nm based on the (002) crystal plane of graphene. Using Equation (12), the interlayer spacing (*d*_*hkl*_) of the prepared G was calculated to be ≈0.36 nm, which is slightly larger than that of standard graphite, but the absence of the (001) peak characteristic of GO indicates that the reduction process was successful. The number of layers of G in this study was estimated to be ≈4 layers. The XRD pattern of CNT was similar to that of G, with a d‐spacing in the (002) crystal plane of ≈0.34 nm.^[^ ^67^ ^]^ Sample F showed sharper peaks and was found to be more crystalline than the other samples, with *L*_*c *_ ≈ 41 nm using the (111) crystal plane. The corresponding d‐spacing for this plane was determined to be 0.80 nm, which is in consistent with TEM results.

The XRD patterns of P25, Bi–BiOBr, Bi–BiOBr–P–GO1, Bi–BiOBr–P–G1, Bi–BiOBr–P–CNT1, and Bi–BiOBr–P–F1 are depicted in Figure 3b. P25 shows the (101), (004), and (020) crystal planes of anatase TiO_2_, and (110), (011), (111), and (211) crystal planes of rutile TiO_2_. Also, the crystal structure of Bi–BiOBr aligns with the expected tetragonal structure for BiOBr (ICSD‐61225‐4) and rhombohedral Bi metal (ICSD‐64703). The composites produced herein contained signals from both P25 and Bi–BiOBr. However, one major difference was the intensity ratio of the (110)/(102) crystal planes in BiOBr, which were similar in the composites but substantially different from the Bi–BiOBr parent material. In the composites, the intensity of the (102) crystal plane was higher than that of the (110) crystal plane. A similar intensity ratio was observed in our previous study of the Bi–BiOBr–P composite.^[^ ^13^ ^]^ None of the composites exhibited distinct peaks of their carbon‐containing materials, likely due to their low loading levels (1 wt%).

**Figure 4:**
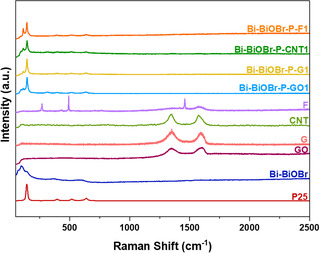
Raman spectra of P25, Bi–BiOBr, GO, G, CNT, F, P25, Bi–BiOBr, Bi–BiOBr–P–GO1, Bi–BiOBr–P–G1, Bi–BiOBr–P–CNT1, and Bi–BiOBr–P–F1 samples from 50–2500 cm^−1^.

**Figure** **4** shows the Raman spectra of GO, G, CNT, F, P25, Bi–BiOBr, Bi–BiOBr–P–GO1, Bi–BiOBr–P–G1, Bi–BiOBr–P–CNT1, and Bi–BiOBr–P–F1. GO shows two distinct peaks at 1350 and 1597 cm^−1^, corresponding to the D and G band, respectively. The G band, which typically appears around 1560 cm^−1^, signifies the pristine double‐bonded sp^2^ carbon in the graphene lattice. Conversely, the D band, observed prominently at ≈1350 cm^−1^, indicates sp^3^ defects within the sp^2^ lattice.^[^ ^44^ ^]^ The ratio between the intensities of the D and G bands (I_D_/I_G_ ratio) is commonly used to assess the degree of disorder in a carbon structure. The obtained I_D_/I_G_ ratio of the synthesized GO is in close agreement with the disorder levels reported for GO produced using the Staudenmaier method.^[^ ^68^ ^]^ The I_D_/I_G_ ratio of the synthesized GO was found to be ≈0.85, signifying the abundance of sp^3^ defects within the sp^2^ lattice. The Raman spectrum of sample G reveals a significantly lower I_D_/I_G_ ratio of 0.128. The Raman spectrum of F reveals several peaks at 268, 491, 1461, and 1570 cm^−1^. The peak at 268 cm^−1^ corresponds to the lowest frequency H_g_ squashing mode of buckminsterfullerene, and the peaks at 491 and 1461 cm^−1^ correspond to the fully symmetric A_g_ intramolecular mode.^[^ ^69^, ^70^ ^]^

The Raman spectra of P25 and Bi–BiOBr were reported in our previous study.^[^ ^13^ ^]^ The Raman spectrum of P25 features prominent peaks at 147, 399, 515, and 635 cm^−1^, corresponding to the E_g_, B_1g_, A_1g_ + B_1g_, and E_g_ vibrational modes of the anatase phase, respectively.^[^ ^13^, ^71^, ^72^ ^]^ In the spectrum of Bi–BiOBr, peaks at 67, 96, and 110 cm^−1^ are attributed to the A_1g_ (internal Bi–Br stretching) mode, while the peak at 142 cm^−1^ corresponds to the E_1g_ (internal Bi–Br stretching) mode. Additionally, the peak at 370 cm^−1^ is associated with the B_1g_ mode, which involves the motion of oxygen atoms.^[^ ^73^, ^74^, ^75^ ^]^ The Raman spectra of Bi–BiOBr–P–GO1, Bi–BiOBr–P–G1, Bi–BiOBr–P–CNT1, and Bi–BiOBr–P–F1 include peaks from both P25 and Bi–BiOBr parent materials. No significant shifts in the peaks are observed compared to their parent materials. Focusing on the characteristic peaks of the carbon‐based material between 1000 and 2000 cm^−1^, the Raman spectra of Bi–BiOBr–P–GO1, Bi–BiOBr–P–G1, Bi–BiOBr–P–CNT1, and Bi–BiOBr–P–F1 are shown in Figure S4 (Supporting Information) alongside the spectra of the GO, G, CNT, and F parent materials. The intensities of the D and G bands are approximately equal in the composites to their parent materials, suggesting that the synthesis conditions in forming the composite did not significantly alter any of the carbon‐based materials used herein.

### 3.5 ATR–FTIR Spectroscopy

**Figure 5:**
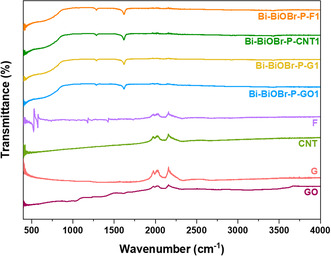
ATR–FTIR spectra of GO, G, CNT, F, Bi–BiOBr–P–GO1, Bi–BiOBr–P–G1, Bi–BiOBr–P–CNT1, and Bi–BiOBr–P–F1 samples from 400 to 4000 cm^−1^.

The FTIR spectra of Bi–BiOBr–P–GO1, Bi–BiOBr–P–G1, Bi–BiOBr–P–CNT1, and Bi–BiOBr–P–F1 were compared to neat GO, G, CNT, and F, respectively, to understand the chemical differences between the inorganic and carbon‐based parts of the composites (**Figure** **5**). In the FTIR spectrum of GO the peaks at 1042, 1221, and 1615 cm^−1^ were ascribed to C—OH, O=C—C, and conjugated C=C ring stretching modes, respectively.^[^ ^76^ ^]^ The FTIR spectra of both G and CNT did not show any peaks attributable to oxygen functional groups. And the FTIR spectrum of F shows several narrow peaks that represent C—C bonds at 1200 and 1450 cm^−1^.^[^ ^77^ ^]^ The FTIR spectra of Bi–BiOBr–P–GO1, Bi–BiOBr–P–G1, Bi–BiOBr–P–CNT1, and Bi–BiOBr–P–F1 show some peaks from 400 to 700 cm^−1^ that can be related to the A_2u_‐type valent symmetrical vibrations of the Bi—O bond (505 cm^−1^),^[^ ^78^ ^]^ and the Ti—O stretching modes and Ti—O—Ti bridging stretching modes, which are typically observed between 400 and 700 cm^−1^.^[^ ^79^, ^80^, ^81^ ^]^ Also, a significant peak at ≈1620 cm^−1^ can be assigned to the deformative vibration of Ti—OH stretching mode.^[^ ^78^ ^]^ There are no peaks that indicated any significant change in the carbon‐based material upon forming a composite with Bi–BiOBr–P.

### 3.6 UV–Visible DRS

**Figure 6:**
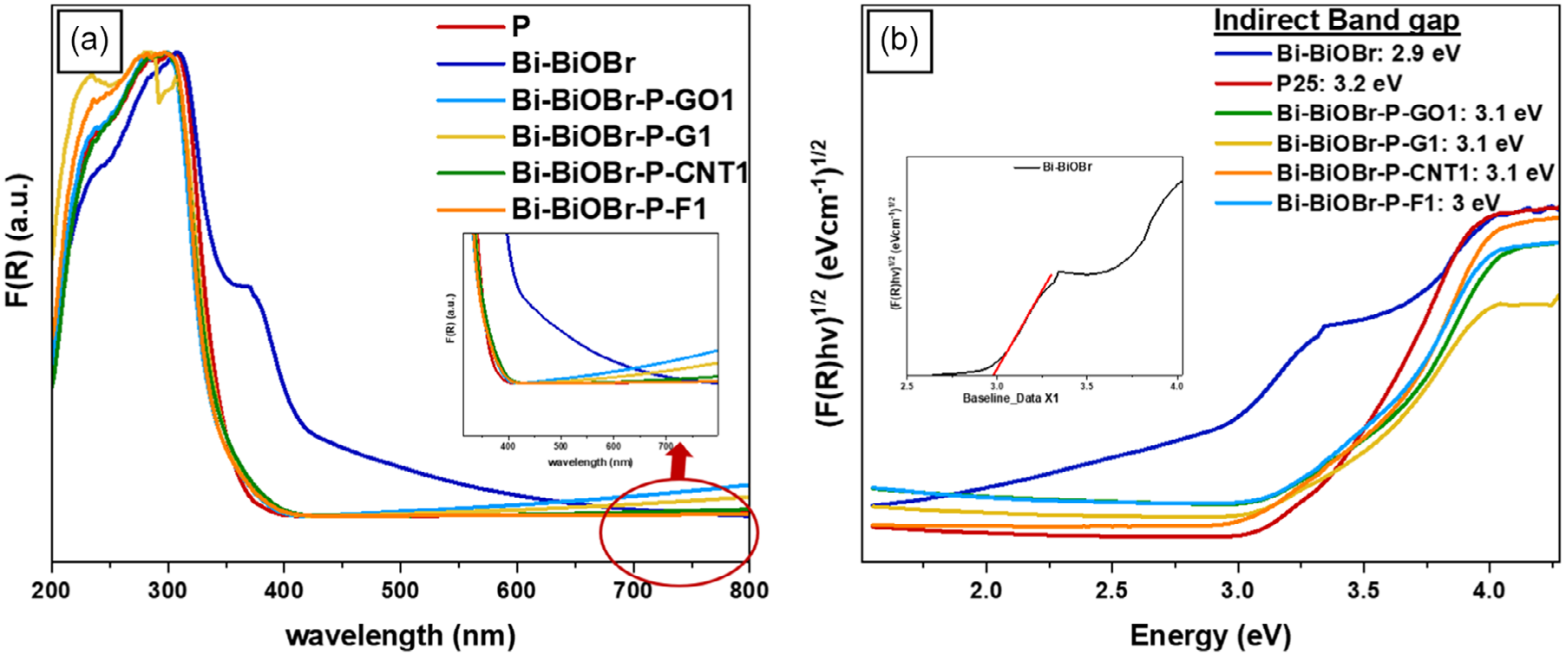
Optical properties of the composites and parent materials. a) UV–visible absorption spectra of Bi–BiOBr, P25, Bi–BiOBr–P–GO1, Bi–BiOBr–P–G1, Bi–BiOBr–P–CNT1, and Bi–BiOBr–P–F1 obtained by converting the measured values of diffuse reflectance to relative absorbance using the Kubelka–Munk relation. b) Tauc plots of Bi–BiOBr, P25, Bi–BiOBr–P–GO1, Bi–BiOBr–P–G1, Bi–BiOBr–P–CNT1, and Bi–BiOBr–P–F1, with the indirect bandgap energy stated next to each sample name.

The optical properties of Bi–BiOBr, P25, Bi–BiOBr–P–GO1, Bi–BiOBr–P–G1, Bi–BiOBr–P–CNT1, and Bi–BiOBr–P–F1 composites were investigated using UV–visible DRS. The collected diffuse reflectance data were converted into relative absorption values through the Kubelka–Munk function (Equation (1)). The indirect allowed bandgap energies (n = 0.5) were then determined using the Tauc plot method (Equation (2)),^[^ ^50^, ^51^, ^52^, ^53^, ^54^, ^55^ ^]^ where F(R) was used as a surrogate for the absorption coefficient. After applying a baseline correction, to remove the influence of the Urbach tail states on the bandgap energy derivation, the plots of these variables against photon energy were extrapolated to the energy axis to estimate the bandgap energies (**Figure** **6**). Figure 6b presents the baseline correction and corresponding bandgap determination for Bi–BiOBr as a representative example.^[^ ^82^, ^83^ ^]^

The absorption spectra of the carbon‐based materials are shown in Figure S5, Supporting Information. All carbon‐based samples showed a strong absorption in the UV region, associated with π–π* transition for the C=C bond.^[^ ^84^ ^]^ The GO, G, and CNT samples showed strong broadband absorption across the visible region, whereas the F sample showed a decrease in absorption in the red region of the electromagnetic spectrum. Figure 6 illustrates the absorption behavior of the composites. While Bi–BiOBr exhibits an absorption edge at 440 nm, P25, Bi–BiOBr–P–GO1, Bi–BiOBr–P–G1, Bi–BiOBr–P–CNT1, and Bi–BiOBr–P–F1 show an absorption edge around 400 nm. Bi–BiOBr, P25 and Bi–BiOBr–P have bandgaps of 2.9, 3.2, and 3.1 eV, respectively, as reported in our previous study.^[^ ^13^ ^]^ This suggests that the addition of carbon‐based materials to the composite does not significantly alter the bandgap or visible light absorption. The broad visible light absorption of the Bi–BiOBr sample can be attributed to the formation of metallic and plasmonic Bi, as confirmed by our XRD results.^[^ ^85^ ^]^ The carbon‐based composites showed a lower degree of visible light absorption, which may be due to a lower level of plasmonic Bi formation in these compounds, as indicated by our XRD results. Moreover, the broadband visible absorption seen in the pure carbon materials (Figure S5, Supporting Information) was not seen in the composites, which we attribute to the high dilution of the carbon‐based materials in these composites (1 wt%).

### 3.7 XPS

XPS measurements were conducted on GO, G, CNT, F, Bi–BiOBr, P25, Bi–BiOBr–P–GO1, Bi–BiOBr–P–G1, Bi–BiOBr–P–CNT1, and Bi–BiOBr–P–F1 to analyze their chemical composition and oxidation states; Table S1 (Supporting Information) provides a summary of the binding energies of the key environments and Table S2 (Supporting Information) provides the atomic percentage of each element.

The XPS analysis of Bi–BiOBr and P25 was previously performed in our earlier study.^[^ ^13^ ^]^ The XPS scans of Bi–BiOBr revealed the presence of Br, Bi, O, and C. The Br 3d spectrum displayed two peaks associated with Br^−^ 3d_7/2_ (68.2 eV) and Br^−^ 3d_5/2_ (69.2 eV).^[^ ^13^ ^]^ The O 1s spectrum showed two peaks, indicating Bi—O bonds at 530.05 eV and surface hydroxyl groups or organic C—O bonds at 531.53 eV (adventitious carbon).^[^ ^13^ ^]^ The C 1s spectrum presented peaks for C—C (284.8 eV), C—O (286.37 eV), and C=O (288.46 eV) bonds, which were attributed to adventitious carbon.^[^ ^13^ ^]^ The Bi 4f spectrum exhibited four peaks, indicating the presence of Bi^3+^ 4f_7/2_ (159.16 eV) and Bi^3+^ 4f_5/2_ (164.48 eV), along with Bi^0^ 4f_7/2_ (157.17 eV) and Bi^0^ 4f_5/2_ (162.59 eV).^[^ ^13^ ^]^ While the predominant oxidation state is Bi^3+^, the presence of metallic Bi^0^ states was also confirmed, likely due to the use of ethylene glycol that can act as a reducing agent in the solvothermal reaction at 180 °C, as supported by our XRD and TEM findings. Additionally, in our previous work, the XPS scans of P25 displayed C 1s, O 1s, and Ti 2p environments.^[^ ^13^ ^]^ The O 1s spectrum showed two peaks indicating a Ti—O bond at 530.17 eV and surface‐adsorbed oxygen groups or organic C—O at 531.18 eV. The C 1s scan revealed peaks at 284.8, 286.1, and 289.23 eV, corresponding to C—C, C—O, and C=O bonds attributed to adventitious carbon.^[^ ^13^ ^]^ In the Ti 2p region, the spectra showed Ti^4+^ 2p_3/2_ at 458.93 eV and Ti^4+^ 2p_1/2_ at 464.77 eV.^[^ ^13^ ^]^

Figure S6–S9 (Supporting Information) show the XPS scans of GO, G, CNT, and F with survey, C 1s and O 1s environments, respectively. Typically, graphitic materials exhibit a primary C 1s peak corresponding to C=C bonds, commonly used as a charge reference calibrated to 284.5 eV.^[^ ^86^ ^]^ Due to the highly heterogeneous nature of most carbon materials—containing differing proportions of sp^2^ and sp^3^ carbon—the carbon spectral envelope often becomes quite complex. In this study, when fitting the carbon core‐level peaks for each sample, we concentrate on two main components: the diamond‐like (sp^3^ hybridized) carbon, represented by a Voigt‐like peak centered at 285 eV, and the graphitic (sp^2^ hybridized) carbon, characterized by an asymmetric peak centered at 284.5 eV. The D‐parameter in XPS plays a crucial role in distinguishing between these carbon states during peak fitting. There is a linear relationship between pure sp^2^ and sp^3^ carbons, determined by analyzing the first derivative of the carbon Auger peak, which reflects the relative concentrations of sp^2^ and sp^3^ carbon. The D‐parameter can be readily measured from the Auger peak in the XPS survey spectra, and it allows calculation of the sp^3^/sp^2^ ratio, which should be incorporated into the carbon core‐level peak fitting settings. Further details on the sp^3^/sp^2^ ratio can be found in the works of Morgan et al. and Lascovich et al.^[^ ^87^, ^88^ ^]^ The C 1s scan of GO, G, CNT, and F all show six distinct environments, corresponding to C=C (sp^2^) (284.5 eV),^[^ ^87^ ^]^ C—C (sp^3^) (285 eV),^[^ ^87^ ^]^ C—O, C=O, O—C=O, and π–π* satellites (Table S1, Supporting Information).^[^ ^86^, ^87^, ^89^, ^90^ ^]^ As anticipated, the C 1s spectrum of GO shows a higher abundance (%) of C—C bonds compared to C=C bonds. Furthermore, there is a significantly high oxygen content (%) in the GO samples as compared to the G samples. In contrast, the C 1s spectra of G, CNT, and F display a higher proportion of C=C bonds relative to C—C bonds, reflecting a preserved and unfunctionalized crystalline structure.

Figure S10–S15 (Supporting Information) show the XPS spectra of the Bi–BiOBr–P–GO1, Bi–BiOBr–P–G1, Bi–BiOBr–P–CNT1, Bi–BiOBr–P–F1, P25, and Bi–BiOBr with Br 3d, Bi 4f, O 1s, C 1s, and Ti 2p environments, respectively. The C 1s spectrum of Bi–BiOBr–P–GO1 shows a higher proportion of C=C bonds relative to C—C bonds, indicating that the GO underwent a partial reduction during the synthesis of the composite. No significant changes in the binding energies of Br 3d, Bi 4f, O 1s, C 1s, and Ti 2p are observed among Bi–BiOBr–P–GO1, Bi–BiOBr–P–G1, Bi–BiOBr–P–CNT1, and Bi–BiOBr–P–F1 compared to their parent materials, as demonstrated for the Bi 4f environment in Figure S16, Supporting Information.

The low‐binding‐energy XPS analysis was performed to determine the valence band maximum (E_VBM_) energy levels of Bi–BiOBr, P25, Bi–BiOBr–P–GO1, and GO relative to their respective Fermi levels (E_F_) (Figure S17, Supporting Information). The measured E_VBM_ values were 2.16, 2.43, 2.11, and 2.86 eV, respectively. These values were then adjusted to the normal hydrogen electrode (NHE) scale using previously reported E_F_ versus NHE data. According to Wu et al.^[^ ^91^ ^]^ the E_F_ potential of BiOBr, with dominant (010) planes comparable to the solvothermally synthesized composites in this study, was reported as 0.6 V versus NHE (pH = 0). Similarly, the E_F_ of P25 was reported to be −0.11 V versus NHE (pH = 0).^[^ ^92^, ^93^ ^]^ The conduction band minimum (E_CBM_) energy levels were calculated using Equation (14), with bandgap (E_g_) values obtained from Tauc plots, as summarized in **Table** **4**. Additionally, the low‐binding‐energy XPS results of Bi–BiOBr–P–GO1 were reported concerning the E_F_ of the composite.
(14)

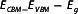




**Table 4:** Band potentials of Bi–BiOBr, P25, and Bi–BiOBr–P–GO1, including the bandgaps (E_g_), valence band maximum (E_VBM_), conduction band minimum (E_CBM_), and Fermi level (E_F_) potentials versus NHE (pH = 0).

Energy Levels	Sample		
	Bi–BiOBr	P25	Bi–BiOBr–P–GO1
E_g_ [V]	2.96 vs. E_F_ (NHE)	3.20 vs. E_F_ (NHE)	3.1 vs E_F_
E_VBM_ [V]	2.76 vs E_F_ (NHE)	2.32 vs E_F_ (NHE)	2.11 vs E_F_
E_CBM_ [V]	−0.2 vs E_F_ (NHE)	−0.89 vs E_F_ (NHE)	−0.99 vs E_F_
E_F_ [V]	+0.60 (NHE) [91]	−0.11 (NHE)^[^ ^92^, ^93^ ^]^	

### 3.8 N_2_‐Sorption Isotherms

**Figure 7:**
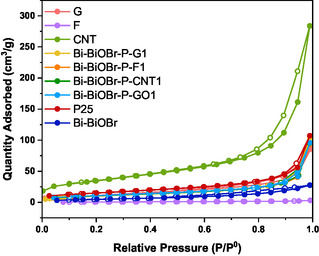
N_2_‐adsorption–desorption isotherms of G, CNT, F, Bi–BiOBr, P25, Bi–BiOBr–P–GO1, Bi–BiOBr–P–G1, Bi–BiOBr–P–CNT1, and Bi–BiOBr–P–F1 obtained at 77 K. Filled symbols represent adsorption and hollow symbols represent desorption.

N_2_‐sorption isotherms were measured at 77 K to determine the surface area and porosity of G, CNT, F, P25, Bi–BiOBr, Bi–BiOBr–P–GO1, Bi–BiOBr–P–G1, Bi–BiOBr–P–CNT1, and Bi–BiOBr–P–F1 (**Figure** **7**). However, the GO sample was not examined because the preparation involved high‐temperature degassing, which could reduce GO to rGO.

The samples show Type II/III isotherms with Type H3/H4 hysteresis loops, typical of materials with narrow, slit‐like pores. The textural properties, including BET area (S_BET_), total pore volume (V_tot_), and micropore volume (V_micro_), are derived from the N_2_‐sorption (77 K) isotherms and presented in **Table** **5**. Based on the results, F is nonporous, with negligible adsorption likely due to the particle accumulation, as observed by TEM and SEM. The second least porous sample is Bi–BiOBr, with an S_BET_ of 18 m^2 ^g^−1^ and a total pore volume of 0.043 cm^3 ^g^−1^. The P25 sample had an S_BET_ of 54 m^2 ^g^−1^, and a total pore volume of 0.166 cm^3 ^g^−1^, showing relatively high porosity likely due to its distinct nanoparticulate size‐distribution. However, Jiang et al. reported a BET surface area of 60.8 m^2^ g^−1^ for P25, produced by the same company as in our study, indicating that slight variations in surface area can occur between different batches.^[^ ^62^ ^]^ The CNT sample showed the highest porosity (S_BET _= 122 m^2^ g^−1^, V_tot _= 0.250 cm^3^ g^−1^). All composites (Bi–BiOBr–P–GO1, Bi–BiOBr–P–G1, Bi–BiOBr–P–CNT1, and Bi–BiOBr–P–F1) showed similar S_BET_ (37–39 m^2 ^g^−1^) and V_tot_ (0.063–0.067 cm^3 ^g^−1^) to G (S_BET_ = 42 m^2 ^g^−1^, V_tot _= 0.072 cm^3 ^g^−1^), except Bi–BiOBr–P–GO1, which has higher V_tot_ (0.149 cm^3 ^g^−1^) and was more mesoporous in nature. In summary, the incorporation of i) G, F or CNT into the composites does not affect the surface area or porosity of the materials, but ii) GO affects the pore volume and structure of the composite (not the surface area).

**Table 5:** Textural properties of Bi–BiOBr, P25, G, CNT, F, Bi–BiOBr–P–GO1, Bi–BiOBr–P–G1, Bi–BiOBr–P–CNT1, Bi–BiOBr–P–F1 as derived from N_2_‐sorption isotherms (77 K), including BET surface area (S_BET_), total pore volume (V_tot_), micropore volume (V_micro_), and ratio of micropore volume to total pore volume (V_micro/tot_).

Sample	S_BET_ [m^2 ^g^−1^]	V_tot_ [cm^3 ^g^−1^]	V_micro_ [cm^3 ^g^−1^]	V_meso_ [cm^3 ^g^−1^]
G	42	0.072	0.033	0.039
F	2	0.003	0.002	0.001
CNT	122	0.250	0.099	0.151
Bi–BiOBr	18	0.043	0.018	0.025
P25	54	0.166	0.041	0.125
Bi–BiOBr–P–GO1	39	0.149	0.029	0.120
Bi–BiOBr–P–G1	37	0.065	0.031	0.034
Bi–BiOBr–P–CNT1	37	0.067	0.031	0.036
Bi–BiOBr–P–F1	38	0.063	0.032	0.031

### 3.9 PL Spectroscopy and TAS Analysis

In Figure S18, Supporting Information, the PL emission spectra of GO, G, CNT, and F, along with their composites with Bi–BiOBr–P, are shown when excited with a 375 nm laser. In agreement with previous literature, TiO_2_ exhibits very weak PL emission, peaking around 400 nm, while Bi–BiOBr shows a much stronger and redshifted emission peaking around 480 nm, consistent with its smaller bandgap measured by UV–vis spectroscopy.

All carbonaceous materials exhibit peak emission between 420–480 nm, except for F, which emits around 700–800 nm, as expected. Notably, Bi–BiOBr–P–GO1, Bi–BiOBr–P–G1, Bi–BiOBr–P–CNT1, and Bi–BiOBr–P–F1 display a PL peak (Figure S18, Supporting Information) at the same wavelength as Bi–BiOBr but with a slightly reduced low‐energy shoulder in CNT‐ and F‐doped composites and a strong reduction in GO‐ and G1‐doped composites.

Since excitation at 375 nm primarily excites Bi–BiOBr (as P25 absorbs poorly at this wavelength and carbonaceous additives constitute only a small fraction (≈1%), this indicates charge transfer from Bi–BiOBr to either TiO_2_ or the carbonaceous materials, with particularly strong charge transfer in Bi–BiOBr–P–GO1 and Bi–BiOBr–P–G1 composites. Furthermore, both Bi–BiOBr–P–GO1 and Bi–BiOBr–P–G1 composites exhibit a notable high‐energy shoulder around 420 nm, which is absent in Bi–BiOBr but present in the GO and G1 emission spectra and redshifted from the TiO_2_ emission peak. This suggests that charge transfer to GO or G1 is the more likely mechanism, with the effect being particularly strong in the Bi–BiOBr–P–GO1 composite.

Finally, the Bi–BiOBr–P–GO1 (Figure S18f, Supporting Information) composite exhibits significantly lower PL emission intensity than the other composites, further suggesting the highest degree of charge transfer in this system.

Transient absorption spectra of GO, G, CNT, F, Bi–BiOBr, P25, Bi–BiOBr–P–GO1, Bi–BiOBr–P–G1, Bi–BiOBr–P–CNT1, and Bi–BiOBr–P–F1 at 10 μs, 100 μs, 1 ms, and 10 ms after 355 nm laser pulse excitation (6 ns pulse width, 0.67 Hz, ≈220 μJ cm^−2^ per pulse) at probe wavelengths from 600 to 1100 nm are shown in Figure S19 (Supporting Information). The optical density of Bi–BiOBr–P–GO1, Bi–BiOBr–P–G1, Bi–BiOBr–P–CNT1, and Bi–BiOBr–P–F1 composites show significant changes compared to the parent material in each composite. While GO, G, and F exhibit similar optical density changes, with maximum absorption predominantly observed at 700 nm (except at 1 ms after the laser pulse, where the peak shifts to 600 nm), CNT displays a distinct behavior. In CNT, the maximum absorption occurs between 700 and 900 nm, with a similar shift to 600 nm at 1 ms after the pulse. Bi–BiOBr shows stronger absorption in the green region and weaker absorption in the near‐infrared (Figure S19, Supporting Information). P25 and Bi–BiOBr–P/C‐based composites displayed maximum absorption at around ≈700 nm, with weaker NIR absorption compared to Bi–BiOBr.

Figure S20 (Supporting Information) shows the transient absorption decay kinetics of GO, G, CNT, F, P25, Bi–BiOBr, Bi–BiOBr–P–GO1, Bi–BiOBr–P–G1, Bi–BiOBr–P–CNT1, and Bi–BiOBr–P–F1, from 10 μs to 1 s, after excitation with a 355 nm laser and measured at a probe wavelength of 800 nm. Transient absorption decay kinetics were measured from 10 μs to 1 s after excitation by a 355 nm laser pulse. Figure S21 (Supporting Information) shows the transient absorption decay kinetics of GO, G, CNT, and F samples, from 10 μs to 1 s, after excitation with a 355 nm laser and measured at a probe wavelength of 800 nm. All carbon‐based samples studied herein do not show any photoexcitation behavior themselves on the measured time scale. Typically, an ultrafast TAS is employed to study the response following ultrafast excitation of electrons in GO,^[^ ^94^, ^95^ ^]^ G,^[^ ^96^ ^]^ CNT,^[^ ^97^ ^]^ and F,^[^ ^98^ ^]^ suggesting the excitation and relaxation processes in these carbon‐based materials are likely faster than the time resolution of the instrument used herein.

**Figure 8:**
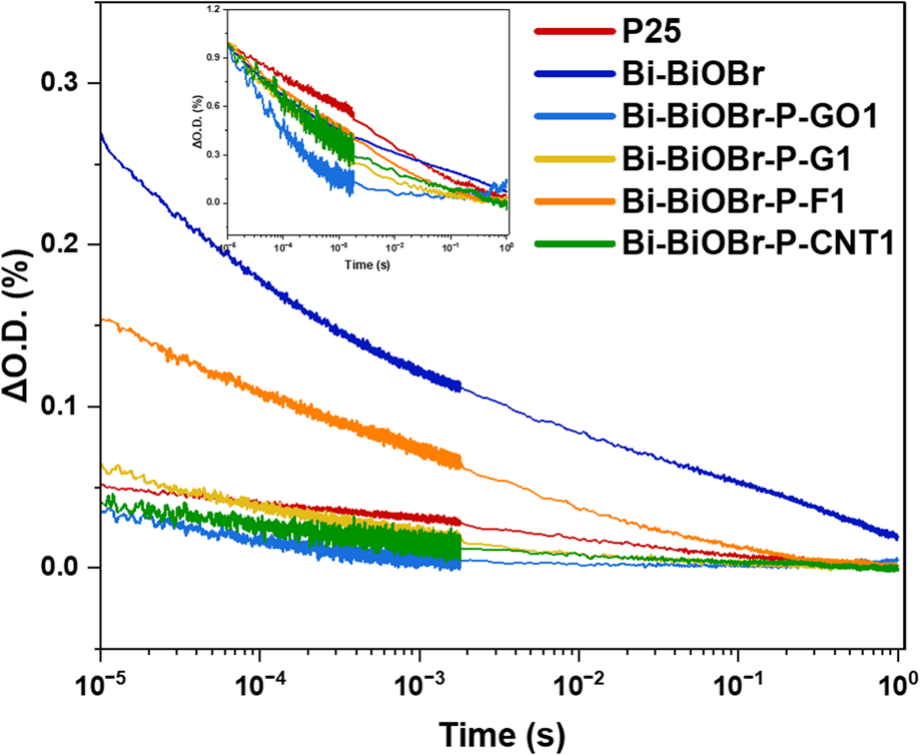
Transient absorption decay kinetics for Bi–BiOBr, P25, Bi–BiOBr–P–GO1, Bi–BiOBr–P–G1, Bi–BiOBr–P–CNT1, and Bi–BiOBr–P–F1 samples measured from 10 μs to 1 s after excitation with a laser (355 nm, 6 ns width, ≈220 μJ cm^−2^ per pulse) and measured at a probe wavelength of 800 nm.

**Figure** **8** shows the transient decay kinetics for Bi–BiOBr, P25, Bi–BiOBr–P–GO1, Bi–BiOBr–P–G1, Bi–BiOBr–P–CNT1, and Bi–BiOBr–P–F1, measured from 10 μs to 1 s after excitation with a laser (355 nm, 6 ns width, ≈220 μJ cm^−2^ per pulse) and measured at a probe wavelength of 800 nm. The decays followed a power‐law function (Equation (15)):

(15) $$f(t)={\text{At}}^{-\alpha }$$

where *f*(*t*) represents the concentration of charge carriers at time *t*, and *A* and α are fitting constants. This type of power‐law behavior arises when ideal bimolecular recombination (characterized by *α* = 1) is interrupted by a multiple‐trapping mechanism. In this mechanism, charge carriers require multiple thermal excitations to reach the band edge, enabling their movement and eventual recombination.^[^ ^99^, ^100^ ^]^ The model was employed to analyze the transient absorption decays observed at the 800 nm probe wavelength, resulting in α values of 0.114, 0.16, 0.43, 0.27, 0.23, and 0.17 for P25, Bi–BiOBr, Bi–BiOBr–P–GO1, Bi–BiOBr–P–G1, Bi–BiOBr–P–CNT1, and Bi–BiOBr–P–F1, respectively. These α values reflect the charge carrier motion and trapping behavior of the samples. The charge carrier lifetimes, represented by t_50%_ (the time required for transient absorption to decay to 50% of its initial value at 10 μs after the laser pulse), were measured as ≈5.4, 0.5, 0.06, 0.2, 0.36, and 0.69 ms for P25, Bi–BiOBr, Bi–BiOBr–P–GO1, Bi–BiOBr–P–G1, Bi–BiOBr–P–CNT1, and Bi–BiOBr–P–F1, respectively, with the decay completing ≈1s after the laser pulse. While P25 exhibited longest‐lived photogenerated charge carriers among the tested samples (see inset of Figure 8), Bi–BiOBr showed the highest initial transient absorption, indicating a higher initial charge carrier population. Except for Bi–BiOBr–P–F1, which demonstrated a higher charge carrier population than P25 (and the second longest‐lived charge carriers), the other composite samples displayed a similar charge carrier population to P25. This suggests that on the pre‐microsecond timescale, charge carrier separation was similar in all the Bi–BiOBr–P–GO1, Bi–BiOBr–P–G1, and Bi–BiOBr–P–CNT1 composites. Of note, the shape of the transient decays in all composites was more similar to that of Bi–BiOBr than that of P25 TiO_2_. This was despite being predominantly composed of P25 TiO_2_ (a 4.4: 1 ratio of Ti: Bi in composition) and was therefore strongly indicative of charge transfer from P25 TiO_2_ to BiOBr sites in the composite.

### 3.10 Photocatalytic Activity

**Figure 9:**
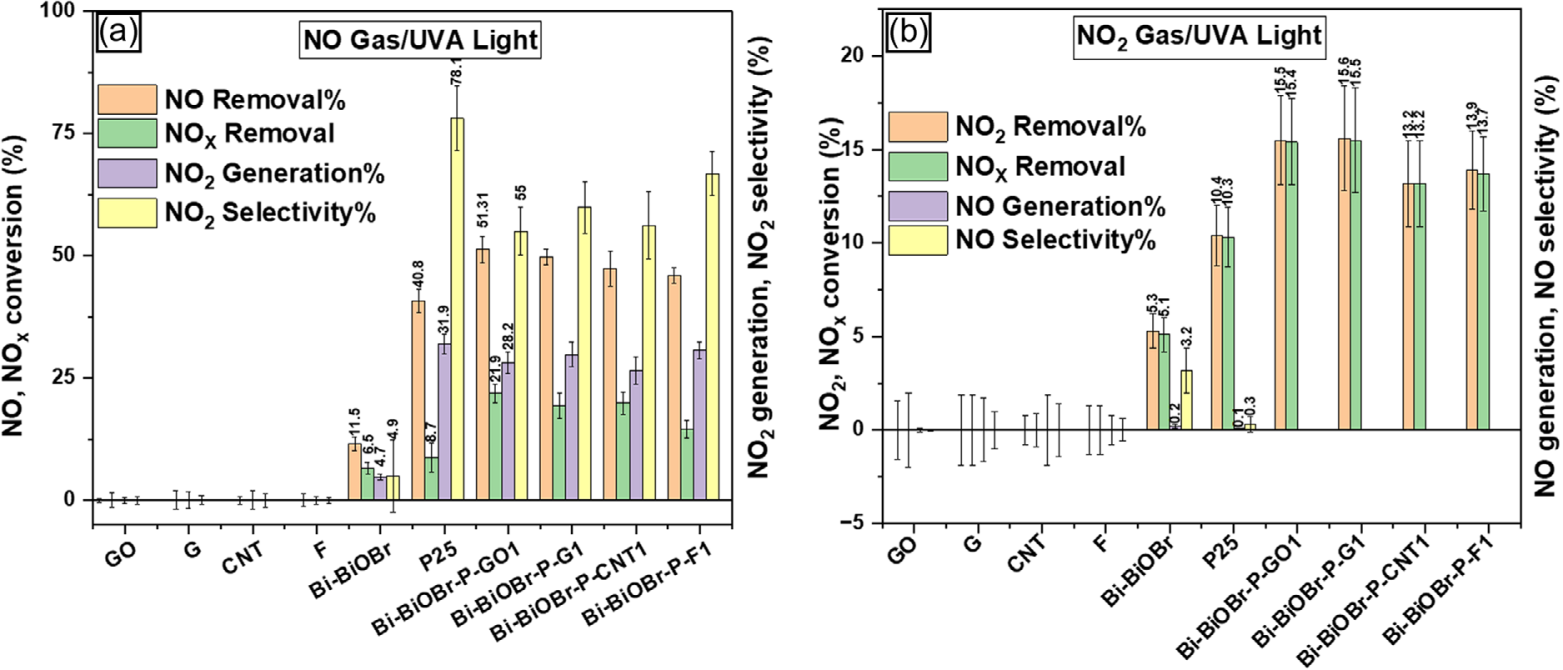
Photocatalytic a) NO removal and b) NO_2_ removal tests for the series of P25, Bi–BiOBr, Bi–BiOBr–P–GO1, Bi–BiOBr–P–G1, Bi–BiOBr–P–CNT1, and Bi–BiOBr–P–F1 samples produced herein summarizing their NO/NO_2_ removal (%), total NO_*x*_ removal (%), NO_2_/NO generation (%), and NO_2_/NO selectivity (%). All samples were examined under UVA light (1.0 mW cm^−2^).

The photocatalytic activity of P25, Bi–BiOBr, Bi–BiOBr–P–GO1, Bi–BiOBr–P–G1, Bi–BiOBr–P–CNT1, and Bi–BiOBr–P–F1 was evaluated against NO and NO_2_ gas under UVA light using ISO 22197‐1:2016 standard protocol. The NO removal results for UVA light are summarized in **Figure** **9**a. The best‐performing composite, Bi–BiOBr–P–GO1 (51.3%), shows higher NO removal activity than P25 (40.8%) and Bi–BiOBr (11.5%). The generation of NO_2_ during photocatalytic NO remediation is a critical factor to consider, as it is between 5 and 25 times more toxic than NO.^[^ ^101^ ^]^ The results show that P25 exhibits a high photocatalytic activity, removing 40.8% of NO. However, this is accompanied by a significant NO_2_ generation of 31.9%, corresponding to a conversion yield of NO to NO_2_ of 78.2%. In contrast, Bi–BiOBr removes 11.5% of NO with only 4.7% NO_2_ generated, showing a lower photocatalytic activity, but reduced production of the more toxic NO_2_, with 40.9% of the removed NO converted to NO_2_. Bi–BiOBr–P–GO1 demonstrates a balanced performance, achieving 51.3% NO removal and generating 28.2% NO_2_, corresponding to a NO‐to‐NO_2_ conversion yield of 54.9%.

The photocatalytic activity was also measured using NO_2_, with the results summarized in Figure 9b. Unlike the parent materials, Bi–BiOBr–P–GO1, Bi–BiOBr–P–G1, Bi–BiOBr–P–CNT1, and Bi–BiOBr–P–F1 do not generate any NO gas during the conversion of NO_2_, indicating that the NO_2_ gas is completely converted to nitrate (NO_3_^−^). The Bi–BiOBr–P–GO1 and Bi–BiOBr–P–G1 samples showed the highest performance among all samples and the parent materials, displaying a NO_x_ removal of 15.35% and 15.5%, respectively. This showed that a synergetic enhancement in performance is seen in reactions with NO_2_ gas for Bi–BiOBr–P–GO1 and Bi–BiOBr–P–G1, with respect to their parent materials. Comparing Bi–BiOBr–P–GO1 and Bi–BiOBr–P–G1 for NO and NO_2_ removal process, since Bi–BiOBr–P–GO1 shows a higher performance for NO removal and the NO_2_ removal was similar, this was deemed the most active material overall.

Under ISO conditions, the performance of Bi–BiOBr–P–GO1 was evaluated over six testing cycles, as shown in Figure S22, Supporting Information. With each successive run, NO_2_ generation gradually increased, while NO removal progressively declined. This indicated that the active sites of the composite were being blocked due to the accumulation of NO_3_^−^ on its surface, which could promote a back reaction leading to the formation of NO and NO_2._^[^ ^102^ ^]^ Notably, after the second cycle, a significant decrease in performance was observed, suggesting that the composite requires washing to restore its activity. After five cycles, the sample was immersed in DI water for 30 min, then dried at 60 °C in an oven for 2 h. When retested, the washed sample exhibited a restoration of activity close to its original level.

#### 3.10.1 The Mechanism of Photocatalytic Action

**Figure 10:**
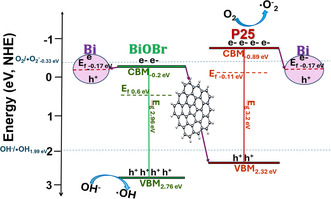
Band energy diagram of the Bi–BiOBr–P–GO1 composite plot alongside the oxidation and reduction potentials of forming reactive oxygen species (ROS) that can drive the oxidation of NO_*x*_.

The suggested mechanism for the photocatalytic action of the Bi–BiOBr–P–GO1 composite is shown in **Figure** **10**. The redox potential of O_2_ reduction to ^•^O_2_^−^ radicals is −0.33 V (vs NHE),^[^ ^103^ ^]^ and as the E_CBM_ of P25 is more negative, it is capable of producing ^•^O_2_^−^ radicals. Alongside this, the oxidation potentials of ^−^OH to ^•^OH and H_2_O to ^•^OH are +1.99 V (vs NHE) and +2.30 V (vs NHE),^[^ ^103^ ^]^ respectively, and both BiOBr and P25 have a more positive E_VBM_ and can produce hydroxyl radicals. However, since the energy offset encourages the accumulation of holes in BiOBr, we believe BiOBr plays a role in producing ^•^OH radicals. While both the Bi‐metal and P25 sites accumulate electrons, just P25 possesses a sufficiently negative E_CBM_ to drive ^•^O_2_^−^ formation; therefore, P25 is responsible for ^•^O_2_^−^ formation. We speculate that the oxidation of NO and NO_x_ is driven by both ^•^OH and ^•^O_2_^−^ radicals in our photocatalyst.^[^ ^104^ ^]^

To better understand the role of ^•^OH and ^•^O_2_^−^ radicals in the photocatalytic activity of our Bi–BiOBr–P–GO1 catalyst, two experiments were designed (Figure S23, Supporting Information). In both NO removal tests, all conditions were kept constant except for RH and the carrier gas used to dilute the NO to 1 ppm. In the first experiment, the NO removal test was conducted with RH reduced to 10% and air as the carrier gas to assess the role of the •OH radical. The results showed 44.17% NO removal, 21.11% NOx removal, and 22.65% NO_2_ generation, indicating a decrease in NO removal from 51.31% (at 50% RH) to 44.17% (at 10% RH). This reduction in NO removal suggests that the ^•^OH radical plays a role in the photooxidation of NO using the Bi–BiOBr–P–GO1 photocatalyst (Figure S23, Supporting Information). In the second experiment, the NO removal test was conducted in the absence of air, with N_2_ gas as the carrier gas, and RH was maintained at 50% to evaluate the role of the ^•^O_2_^−^ radical (Figure S23, Supporting Information). The results showed 3.49% NO removal, 2.94% NOx removal, and 0.5% NO_2_ generation, with NO removal decreasing from 51.31% (using air as the carrier gas) to 3.49% (using N_2_ as the carrier gas). This dramatic reduction in NO removal in the absence of air highlights the critical role of the ^•^O_2_^−^ radical in the photooxidation of NO, compared to the ^•^OH radical.

These findings demonstrate that both ^•^O_2_^−^ and ^•^OH radicals are required for the photooxidation of NO using the Bi–BiOBr–P–GO1 photocatalyst, with the ^•^O_2_^−^ radical playing a distinctly crucial role. The role of GO in Bi–BiOBr–P–GO1 is to accelerate the electron transfer in this Z‐scheme heterojunction and provide many additional active sites to facilitate the oxidation of adsorbed NO and NO_2_ to the catalyst surface to NO_3_^−^.^[^ ^105^, ^106^ ^]^

### 3.11 Comparison with BiOBr and P25 Containing GO in the Literature

Several studies have investigated the photocatalytic activity of carbonaceous material–based composites of TiO_2_ and BiOBr through the photooxidation of NO gas, some of which are listed in **Table** **6**.

**Table 6:** NO_x_ removal photocatalytic activity of photocatalysts containing carbon‐based composites of BiOBr and TiO_2_ from the literature compared with the performance of photocatalysts produced herein.

Material	Pollutant	ISO	RH [%]	Pollutant removal [%]	Light source	Pollutant concentration	Reference
BiOBr/graphene quantum dot	NO gas	No	ns	80.17	1× Xe lamp (300 W)	33–35 ppm	[107]
Ti—C‐dopped BiOBr	NO gas	No	ns	53.6	1× Mercury lamp (<290 nm) (450 W)	1 ppm	[108]
TiO_2_/GO 0.1 wt%	NO gas	Yes	50	52.28	Philips Cleo Compact 15 W lamps	1 ppm	[41]
Black TiO_2_/GO 3 wt%	NO gas	No	ns	50.4	1 × Xe lamp (350 W) (>420 nm)	400 ppm	[109]
TiO_2_/MWCNT 8 wt%	NO gas	Yes	ns	37.1	1× Black UV lamp	1 ppm	[110]
Bi–BiOBr–P–GO1	NO gas	Yes	50	51.31	2× Sankyo (UV lamp), 15 W	1 ppm	This study

ns = not stated.

Qianqian Nie et al. synthesized BiOBr/lignite‐derived graphene quantum dots using a solvothermal method.^[^ ^107^ ^]^ The incorporation of graphene quantum dots significantly enhanced the photocatalytic activity of the composite, increasing NO removal efficiency from 48.44% to 80.17% under a 0.2 L min^−1^ flow of NO gas (33–35 ppm), when irradiated with a 300 W Xe lamp at an intensity of 68.85 mW·cm^−2^.^[^ ^107^ ^]^ Hermawan et al. investigated the effect of Ti and C incorporation into BiOBr for NOx removal under UV light (<290 nm), using the catalyst applied to a 20 × 16 mm substrate. They found that pure BiOBr achieved a NOx conversion efficiency of 47.1%, while the addition of Ti and C increased the activity to 53.6%.^[^ ^108^ ^]^ Tseng et al. prepared TiO_2_/amorphous carbon composites using the impregnation method and evaluated their photocatalytic activity through NOx removal and methyl orange degradation. Trapolis et al. investigated the NOx removal performance of TiO_2_/graphene and TiO_2_/ GO composites, finding that TiO_2_/GO containing 0.1 wt% GO exhibited the highest NOx removal efficiency among the tested samples.^[^ ^41^ ^]^ The photocatalytic tests were conducted under a Philips Cleo Compact UV‐A lamp with an intensity of 10 W m^−2^, a flow rate of 3 L min^−1^, and 50% RH.^[^ ^41^ ^]^ Zhu et al. synthesized black TiO_2_/GO (3 wt%) using a sol–gel method followed by a solvothermal treatment.^[^ ^109^ ^]^ The photocatalytic NO removal activity was evaluated under irradiation from a 350 W Xe lamp (>420 nm) in the presence of 30 wt% H_2_O_2_, with a gas flow rate of 0.1 L min^−1^. The results showed that black TiO_2_ alone achieved a NO removal efficiency of 33.9%, while the addition of 3 wt% GO increased the efficiency to 50.4%, both in the presence of H_2_O_2_. The enhanced photocatalytic performance was attributed to the prolonged lifetime of charge carriers, resulting from the high conductivity of the rGO and the increased surface area of the composite.^[^ ^109^ ^]^ Yen et al. studied the photocatalytic activity of sol–gel‐synthesized TiO_2_/MWCNT composites by evaluating their NO removal performance.^[^ ^110^ ^]^ They investigated the effect of MWCNT incorporation on the photooxidation of NO and found that the addition of 8 wt% MWCNT‐enhanced NO removal efficiency while reducing NO_2_ selectivity. In their experiments, 0.1 g of the composite was used for the NO removal tests.

In this study, the Bi–BiOBr–P–GO1 composite (50 mg) exhibited superior NOx removal performance compared to the parent materials and other synthesized composites, achieving 51.31% NO removal, 21.9% NOx removal, and 28.2% NO_2_ generation under UV light irradiation at an intensity of 1 mW cm^−2^. These results suggest that the composite is a promising photocatalyst for the photooxidation of NOx under ambient conditions, especially in regions with strong natural sunlight, where UV intensities around 1 mW cm^−2^ are commonly observed.

Comparing the performance of the materials studied here with those reported in previous studies is challenging due to significant variations in experimental conditions. Many earlier investigations employed nonstandard reactor geometries, the mass of the sample, the size of the coating on the substrate, light sources and intensities, pollutant concentrations, relative humidities, and gas flow rates that deviate from the ISO 22197‐1:2016 protocol followed in this work.

## 4 Conclusion

In this research, a series of Bi–BiOBr–P–C (C: GO, G, CNT, and F; all at 1 wt%) composites were synthesized using a solvothermal method. To investigate the photocatalytic performance of the samples, they were tested against NO_x_ (NO and NO_2_) gas by ISO protocol (22197‐1:2016).

The Bi–BiOBr–P–GO1 composite exhibited the highest photocatalytic performance compared to the other composites and parent materials. For reactions in NO gas, it showed a combined higher NO_x_ removal rate (21.9%) than its parent materials P25 (8.7%), Bi–BiOBr (6.5%), and GO (0%), which was clear evidence for a synergistic improvement in activity. This was due to an improved selectivity for NO_2_ formation (55.0%) that resulted in significantly lower NO_2_ generation (28.2%). For reactions in NO_2_ gas, it showed a higher NO_x_ removal rate (≈15%) than its parent materials P25 (≈10%), Bi–BiOBr (≈5%), and GO (0%). Moreover, unlike Bi–BiOBr, no NO gas was produced, indicating unity selectivity for oxidizing NO_2_ to NO_3_^−^. The superior performance of Bi–BiOBr–P–GO1 is attributed to several factors. These include the possible chemical bond between Bi and the oxygen functional groups of GO, as suggested by XPS analysis; a higher surface area compared to its parent materials, as revealed by BET measurements; and charge transfer between its component P25 and Bi–BiOBr sites, as demonstrated by DR–TAS results. The reduction in NO_2_ generation during the photooxidation of NO gas using the Bi–BiOBr–P–GO1 composite may also be attributed to an increased affinity for NO_2_ molecules to be absorbed to the surface of GO. This increased affinity would increase the residence time of NO_2_ at the surface of the catalyst, allowing more time for NO_2_ to be further oxidized to NO_3_^−^. Overall, this work shows how carbon‐based materials can improve the photocatalytic activity of materials for NO_x_ remediation and is a promising materials design strategy that requires further exploration in other systems.

## Conflict of Interest

The authors declare no conflict of interest.

## Supporting information

Supplementary Material

## Data Availability

The data that support the findings of this study are available from the corresponding author upon reasonable request.
