# Supplementary material for: Exploring the Influence of Carbonaceous Material on the Photocatalytic Performance of the Composites Containing Bi–BiOBr and P25 TiO2 for NOx Remediation
Source: Chemphyschem. 2025 Jun 19;26(15):e202500237. doi: 10.1002/cphc.202500237 (PMC12321284; doi:10.1002/cphc.202500237)
Supplement: Supplementary file 1 — Supplementary Material [file CPHC-26-e202500237-s001.pdf]

Supporting Information

Exploring the Influence of Carbonaceous Material on the Photocatalytic Performance of the Composites Containing Bi-BiOBr and P25 TiO_2 for NO_x Remediation

Paransa Alimard[1,2,3*], Stanley Cazaly [4], Ioanna Itskou[5], Hanieh Akbari[6], Srinivas Gadipelli[7], Nazila Kamaly[1], Flurin Eisner[4], Andreas Kafizas[1,3*]

[1] *Department of Chemistry, Molecular Science Research Hub, 82 Wood Lane, White City Campus, Imperial College London, London, W12 0BZ, U.K.*

[2] *Science and Solutions for a Changing Planet DTP, Grantham Institute for Climate Change and the Environment, Exhibition Road, South Kensington Campus, Imperial College London, London, SW7 2AZ, U.K.*

[3] *London Centre for Nanotechnology, South Kensington Campus, Imperial College London, London, SW7 2AZ, U.K.*

[4] *School of Engineering and Materials Science, Queen Mary University of London, Mile End Road, London, E1 4NS, U.K.*

[5] *Department of Chemical Engineering, South Kensington Campus, Imperial College London, London, SW7 2AZ, U.K.*

[6] *Department of Physics & Astronomy, University College London, London, WC1E 6BT, U.K.*

[7] *Department of Chemical Engineering, University College London, London, WC1E 6BT, U.K.*

**Co-corresponding authors* = *p.alimard22@imperial.ac.uk and a.kafizas@imperial.ac.uk*

Keywords: bismuth oxybromide (BiOBr); titanium dioxide (TiO_2); carbonaceous composites; nitrogen oxide (NO_x) removal; photocatalysis.

Photographs of the samples

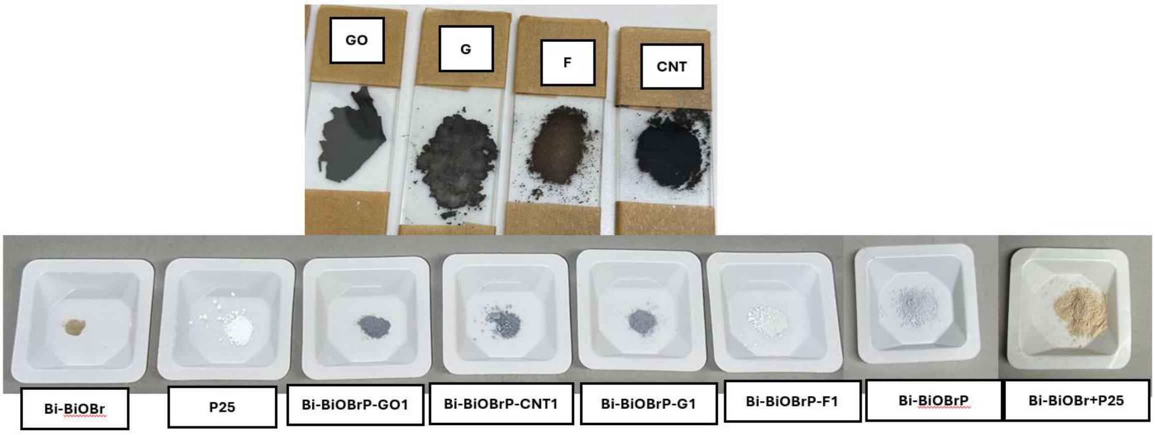

Figure S1. *Photographs of the samples produced and studied in this work. In the top row, GO, G, F, and CNT are shown pressed between two microscope slides and secured with masking tape. From left to right: GO (dark brown), G (black), F (brown), and CNT (black). In the bottom row, from left to right: Bi-BiOBr (brown), P25 (white), Bi-BiOBr-P-GO1 (grey-blue), Bi-BiOBr-P-CNT1 (grey-blue), Bi-BiOBr-P-G1 (grey-blue), Bi-BiOBr-P-F1 (white-grey), Bi-BiOBr-P (white-blue), and Bi-BiOBr mixed with P25 at the same molar ratio as in Bi-BiOBr-P (light brown).*

HR-TEM imaging

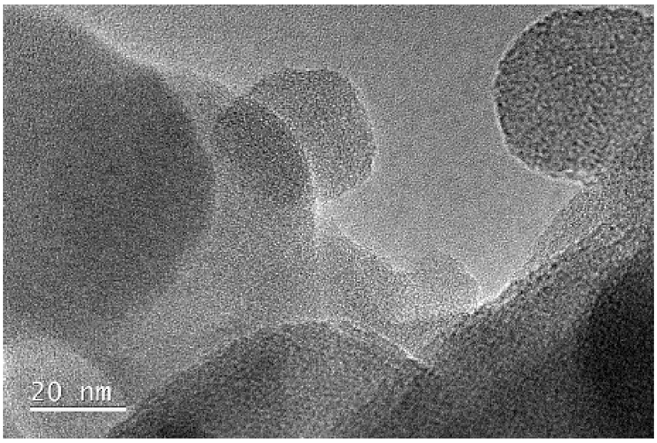

Figure S2*. HR-TEM images of the buckminsterfullerene (F) used in this work.*

XRD and Raman spectroscopy

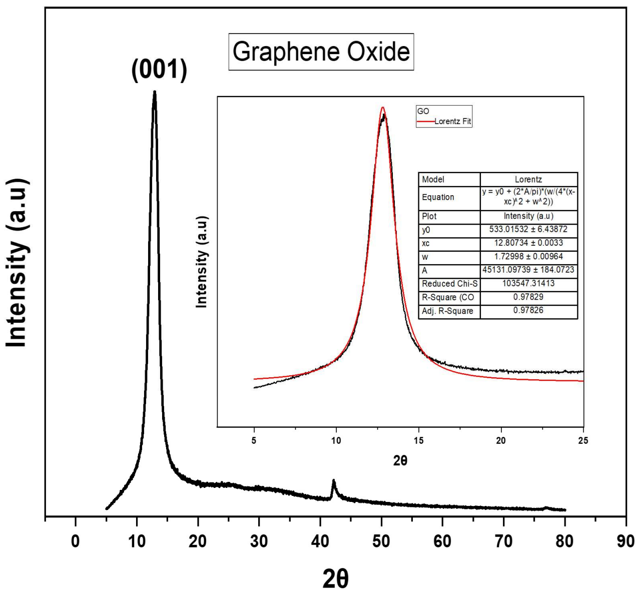

Figure S3. *XRD pattern of graphene oxide (GO) and the FWHM of (001) crystal facet.*

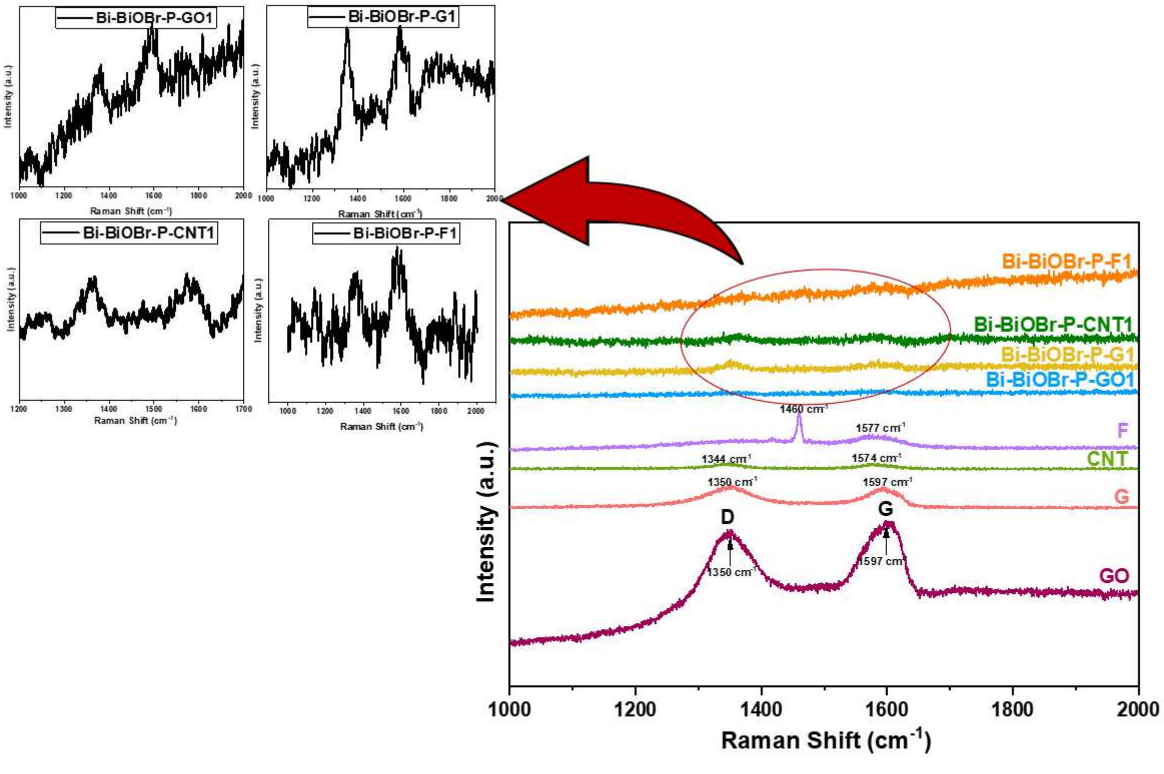

Figure S4. *Raman spectra of GO, G, CNT, F, P25, Bi-BiOBr, Bi-BiOBr-P-GO1, Bi-BiOBr-P-G1, Bi-BiOBr-P-CNT1, and Bi-BiOBr-P-F1 from 1000-2000 cm^{-1}.*

UV-visible diffuse reflectance spectroscopy (DRS)

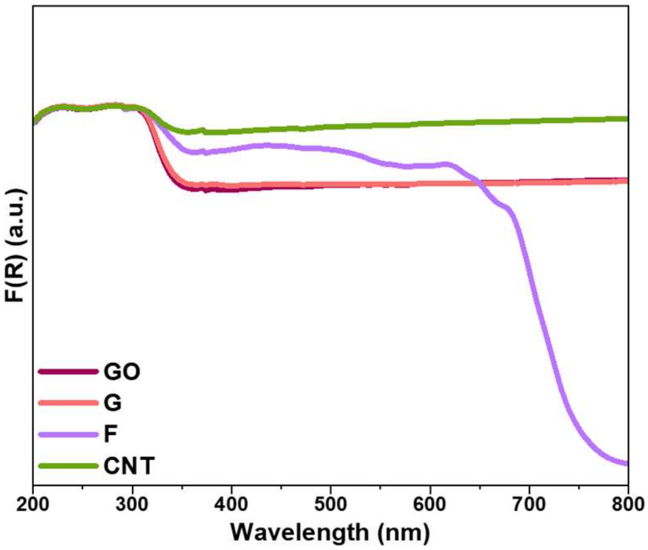

Figure S5. *UV-visible absorption spectra of GO, G, CNT, and F, obtained by converting the measured values of diffuse reflectance to relative absorbance using the Kubelka-Munk relation.*

XPS analysis

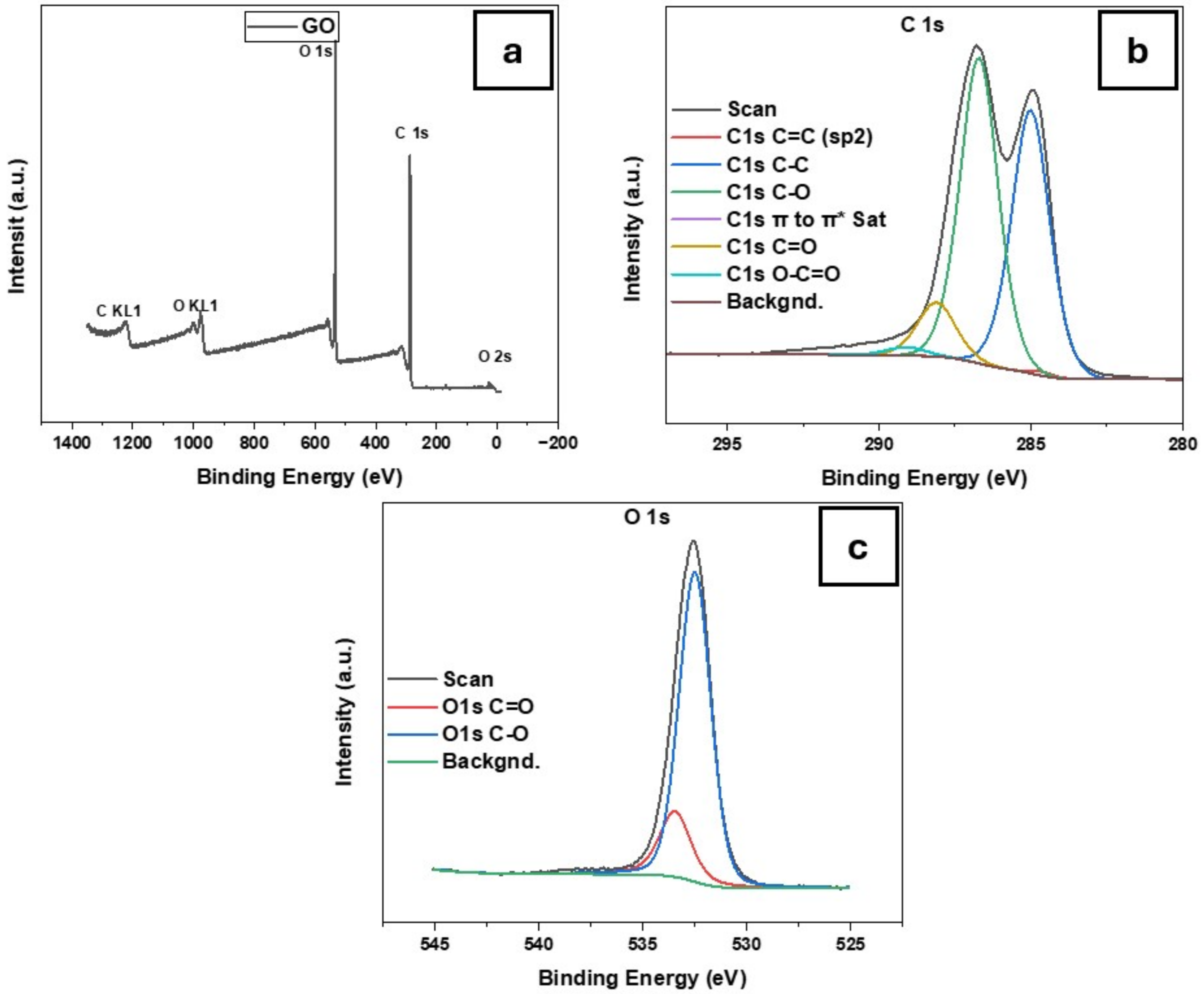

Figure S6. *X-ray photoelectron spectra and fittings of the a) survey, b) C 1s, and c) O 1s, binding energy environments of GO.*

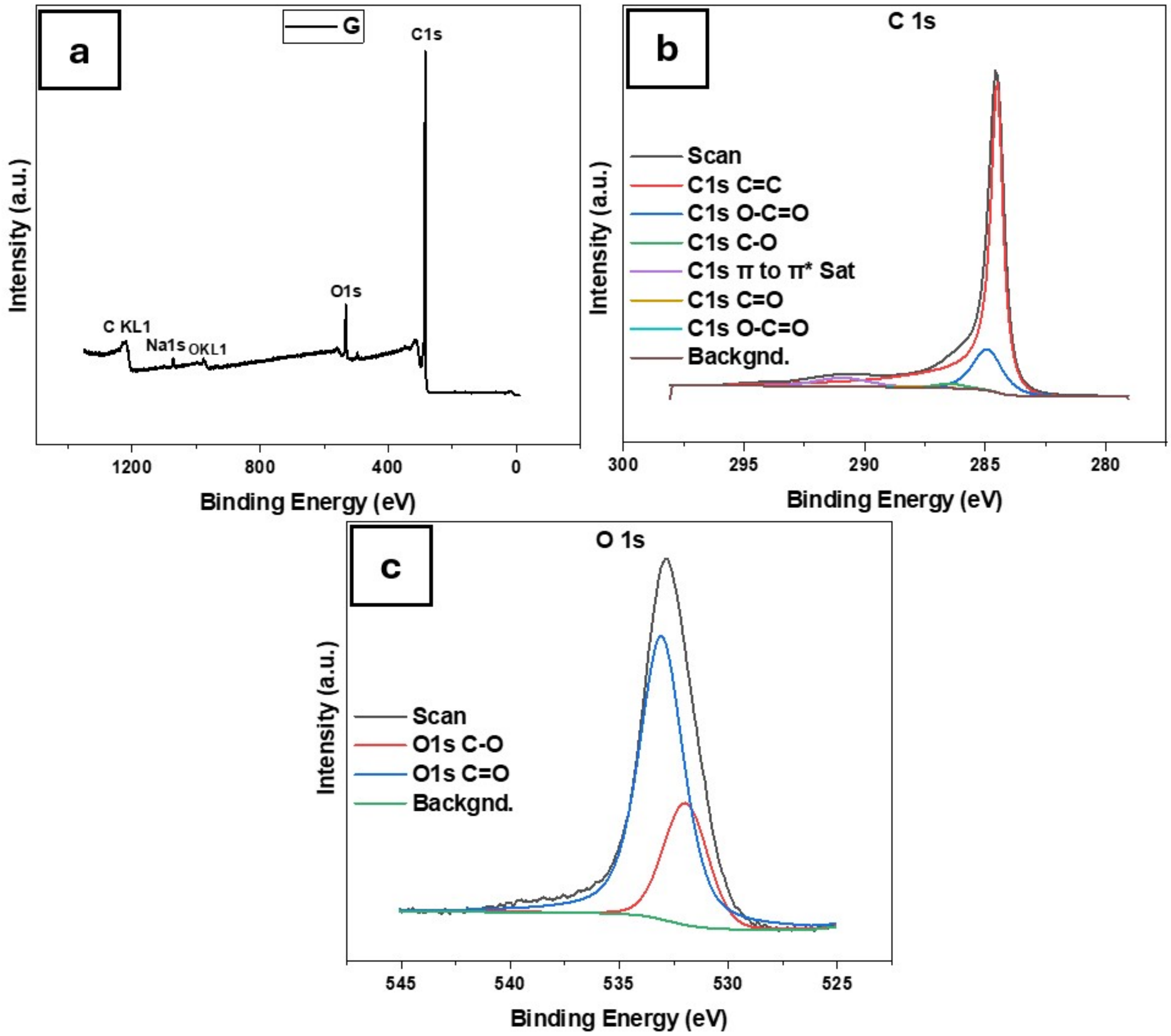

Figure S7. *X-ray photoelectron spectra and fittings of the a) survey, b) C 1s, and c) O 1s, binding energy environments of G.*

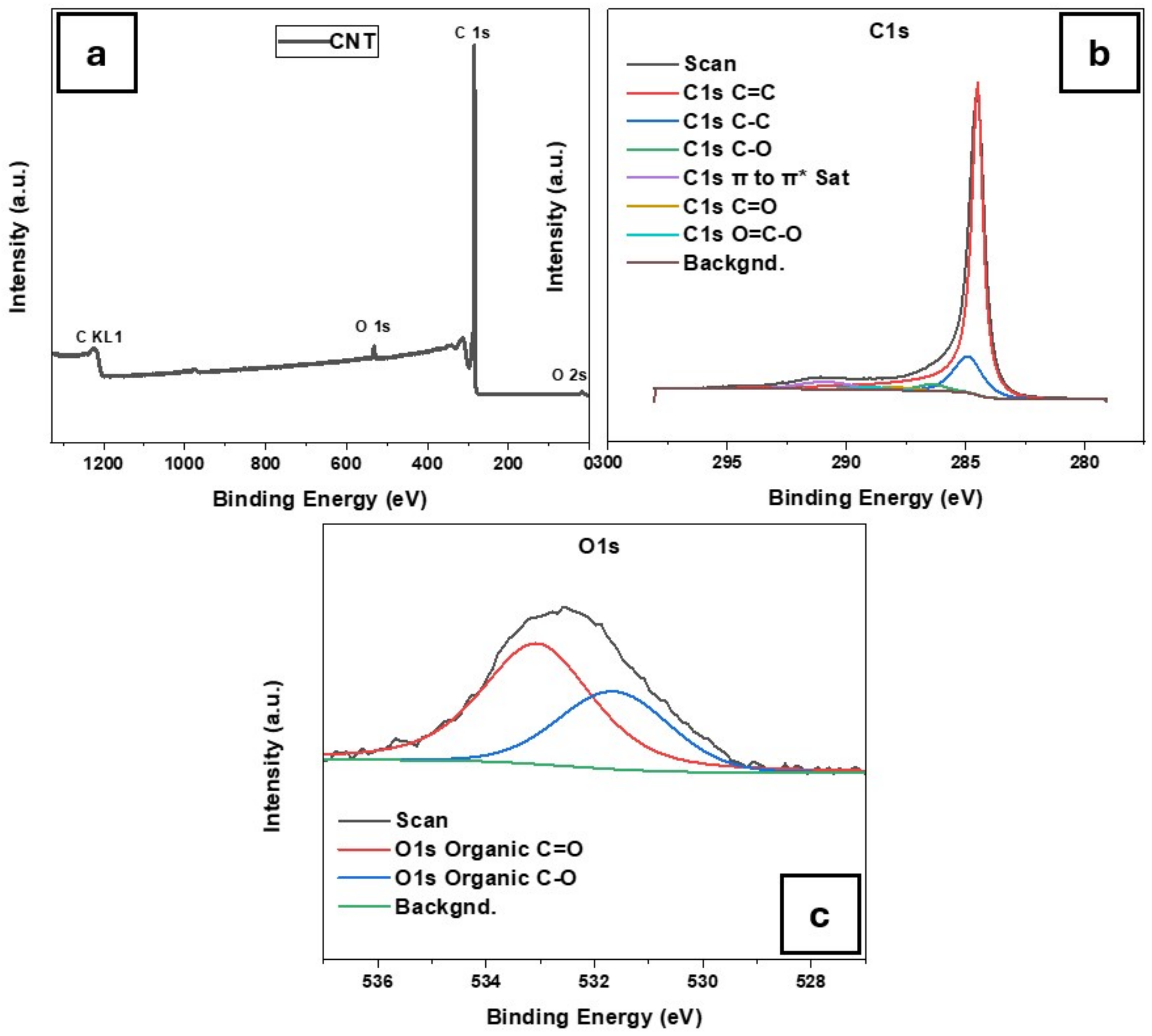

Figure S8. *X-ray photoelectron spectra and fittings of the a) survey, b) C 1s, and c) O 1s, binding energy environments of CNT.*

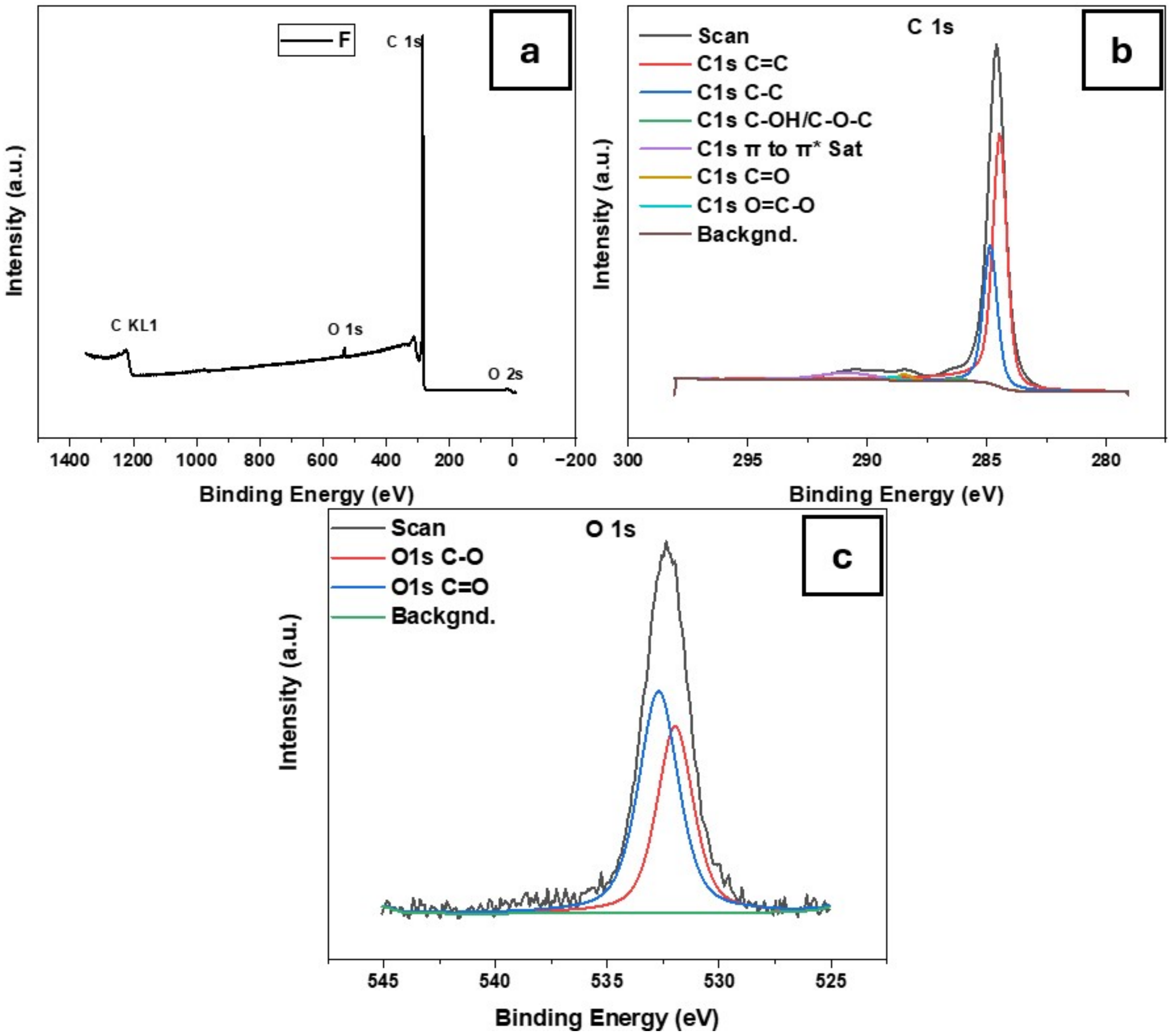

Figure S9. *X-ray photoelectron spectra and fittings of the a) survey, b) C 1s, and c) O 1s, binding energy environments of F.*

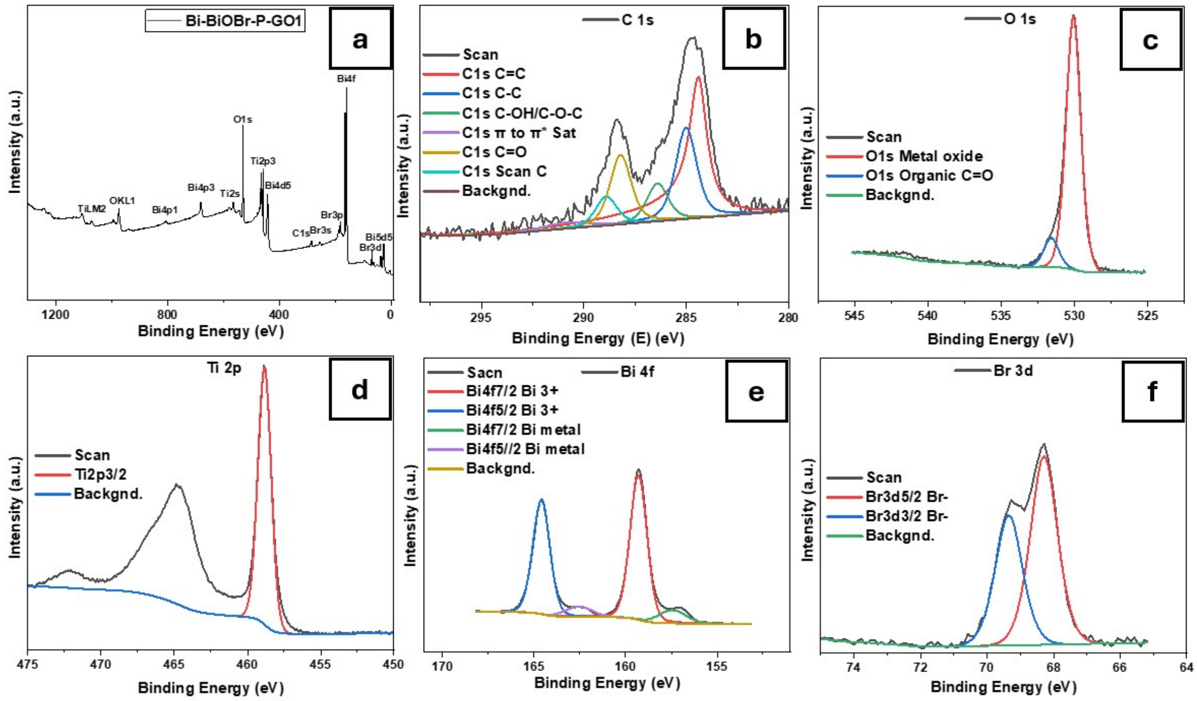

Figure S10. *X-ray photoelectron spectra and fittings of the a) survey, b) C 1s, c) O 1s, d) Ti 2p, e) Bi 4f, and f) Br 3d binding energy environments of Bi-BiOBr-P-GO1.*

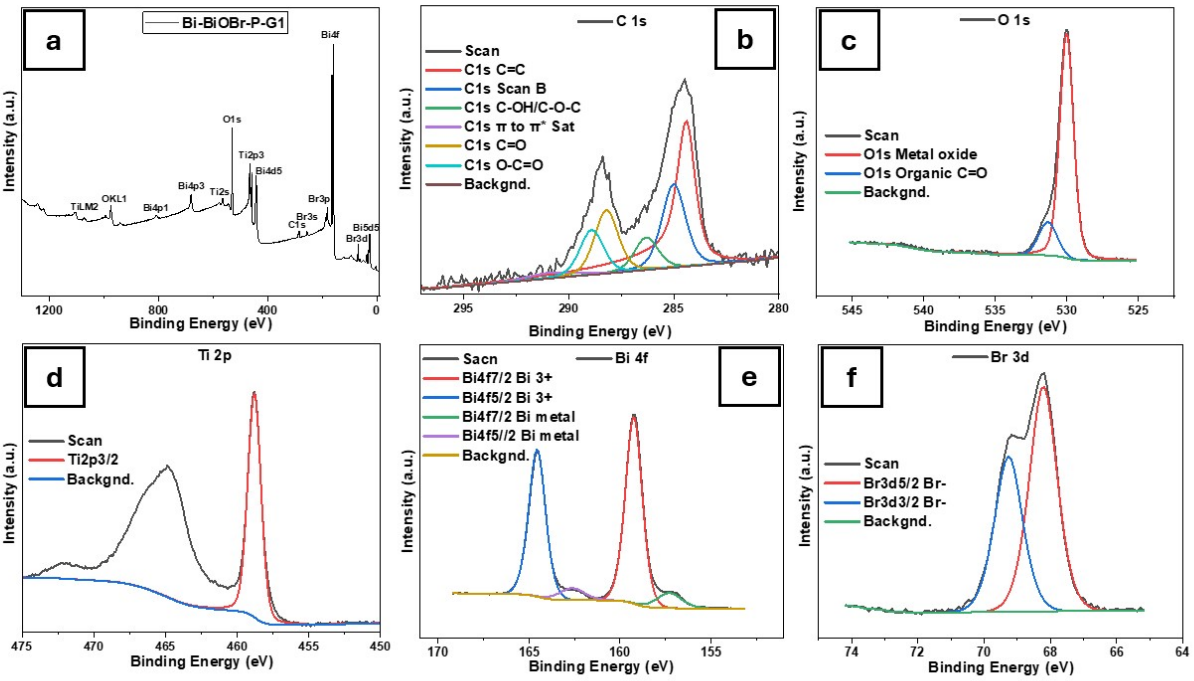

Figure S11. *X-ray photoelectron spectra and fittings of the a) survey, b) C 1s, c) O 1s, d) Ti 2p, e) Bi 4f , and f) Br 3d binding energy environments of Bi-BiOBr-P-G1.*

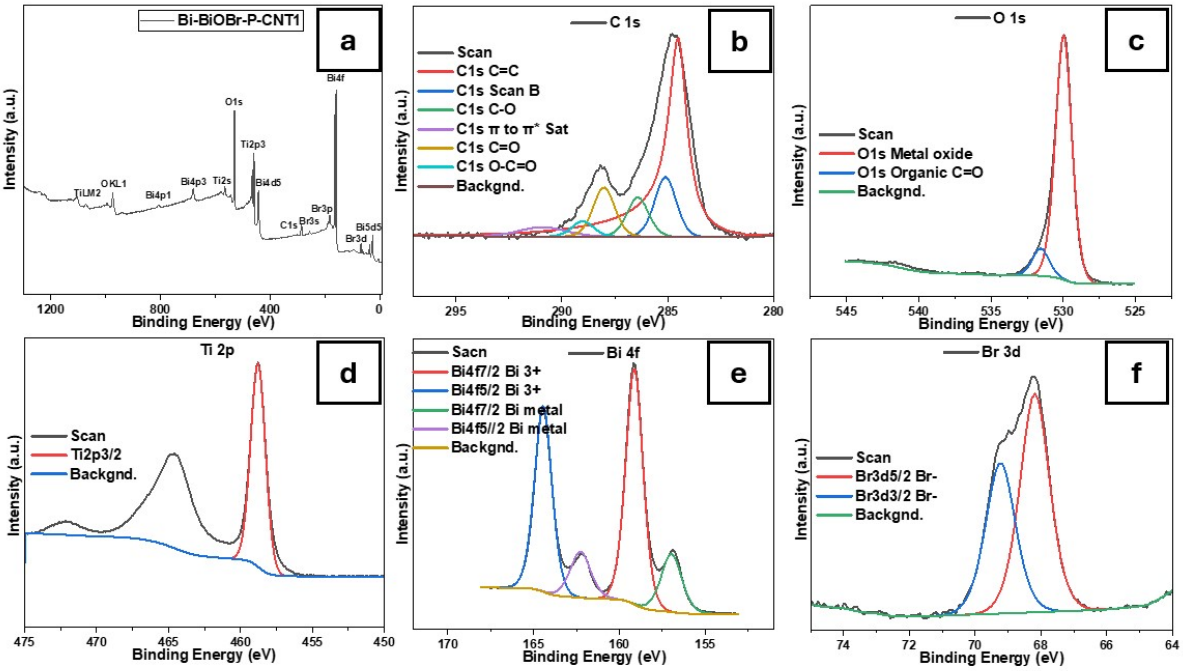

Figure S12. *X-ray photoelectron spectra and fittings of the a) survey, b) C 1s, c) O 1s, d) Ti 2p, e) Bi 4f and f) Br 3d binding energy environments of Bi-BiOBr-P-CNT1.*

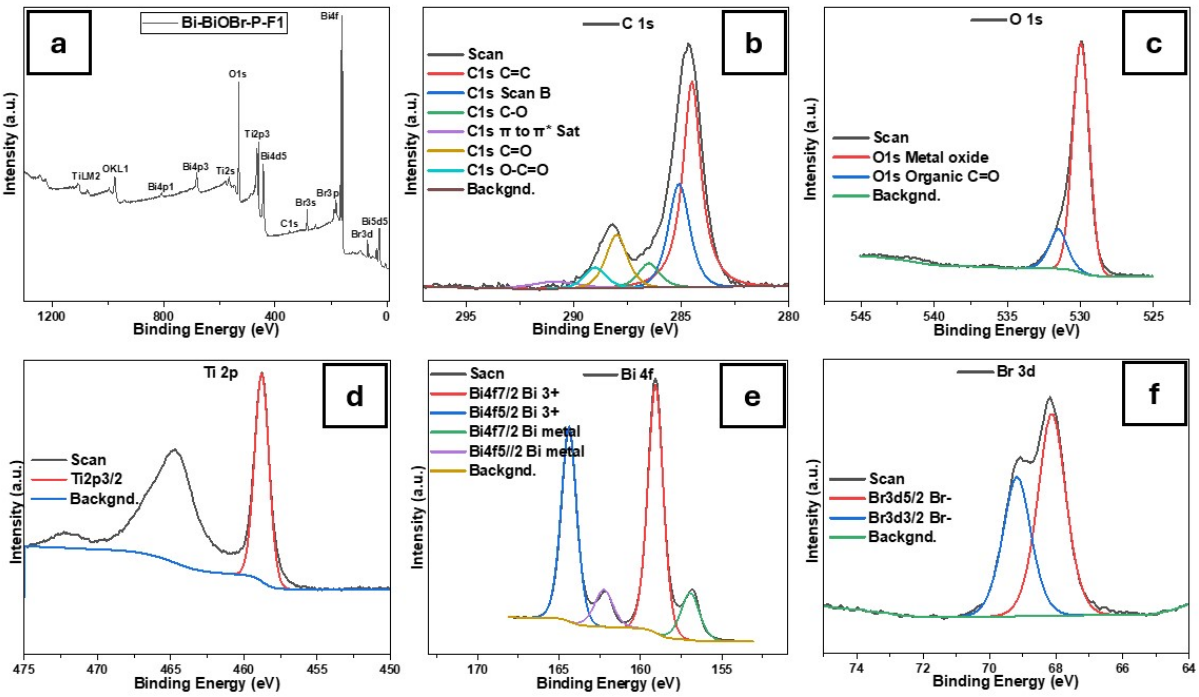

Figure S13. *X-ray photoelectron spectra and fittings of the a) survey, b) C 1s, c) O 1s, d) Ti 2p, e) Bi 4f and f) Br 3d binding energy environments of Bi-BiOBr-P-F1.*

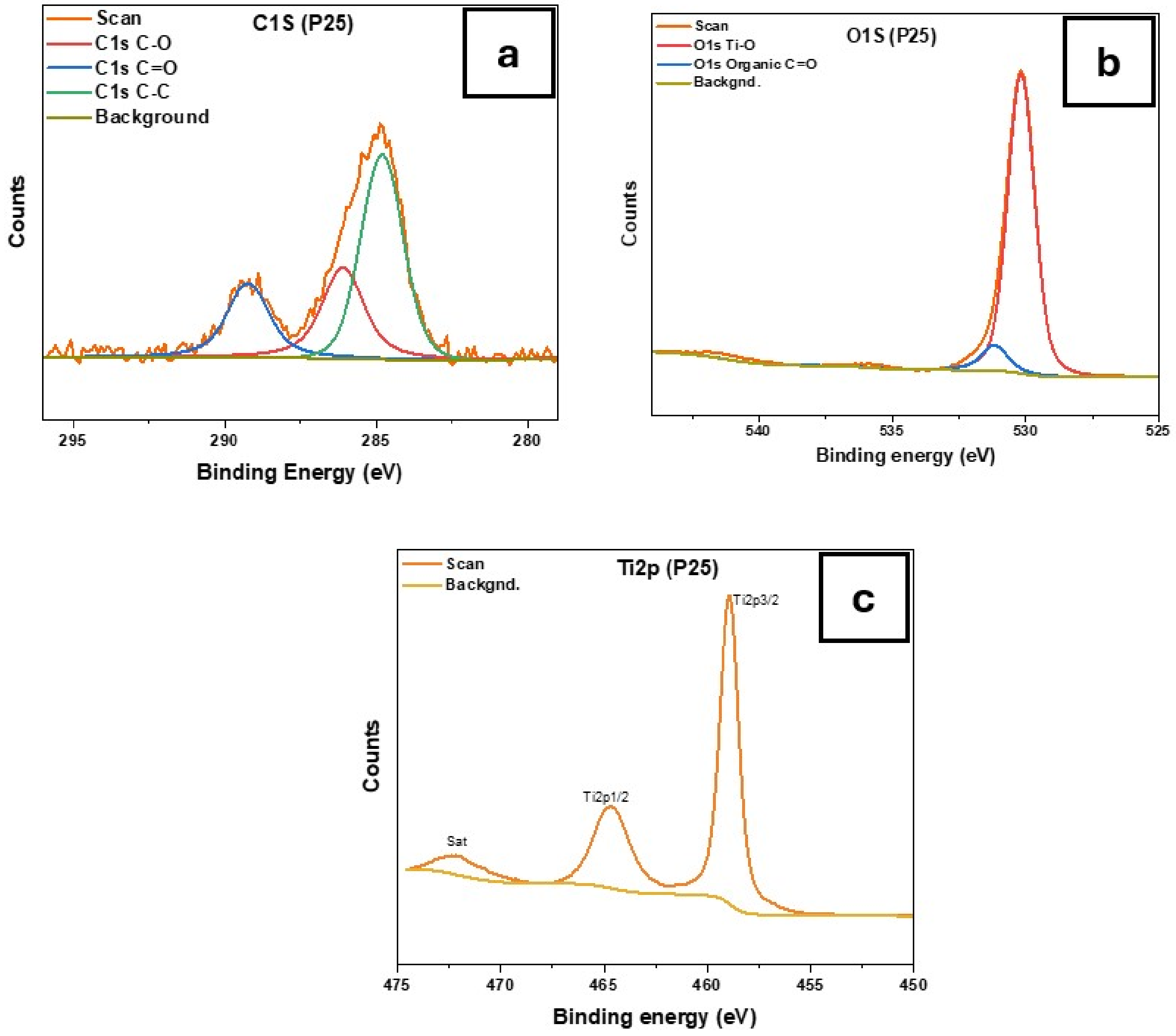

Figure S14. *X-ray photoelectron spectra and fittings of the a) C 1s, b) O 1s, and d) Ti 2p binding energy environments of P25.*

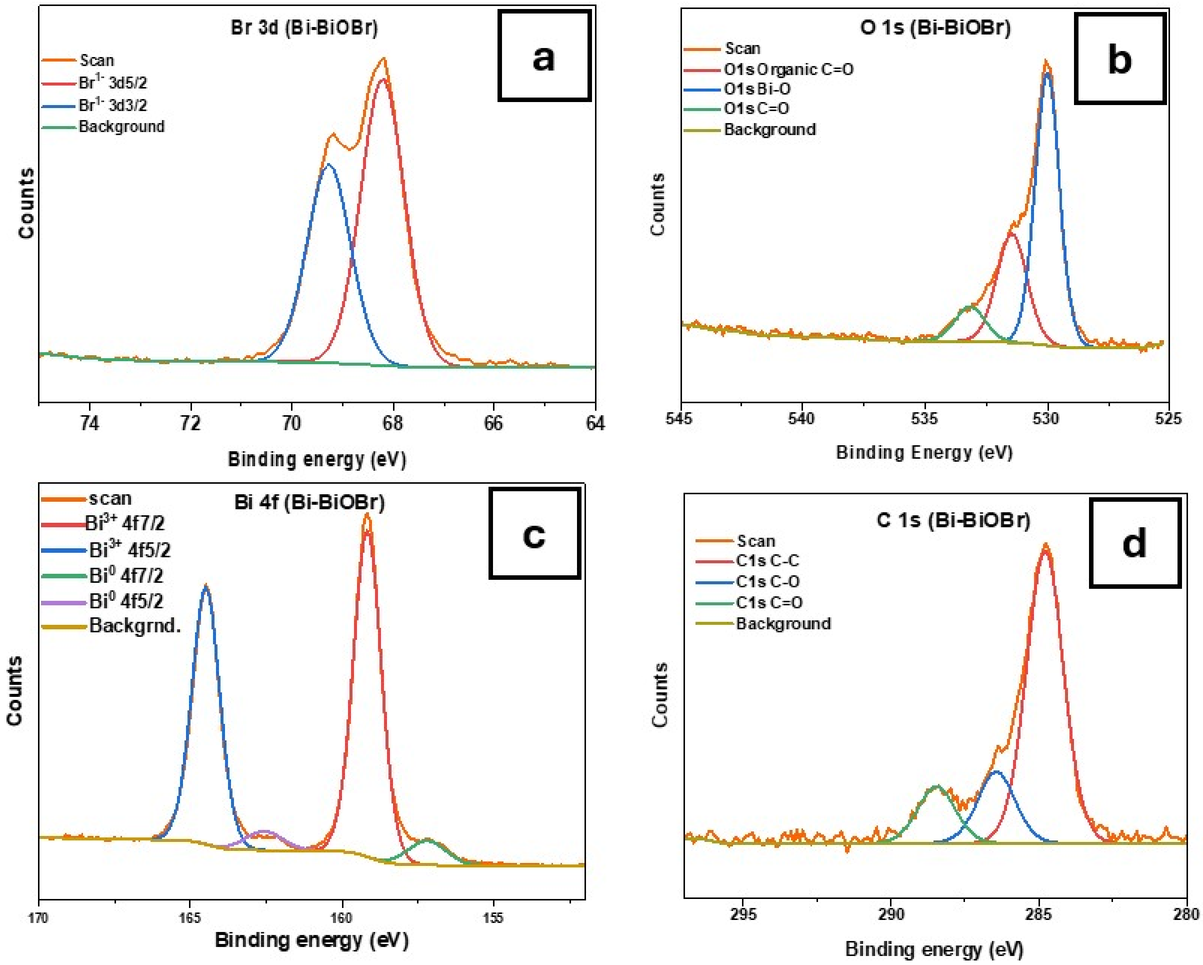

Figure S15. *X-ray photoelectron spectra and fittings of the a) Br 3d, b) O 1s, c) Bi 4f, and d) C 1s binding energy environments of Bi-BiOBr.*

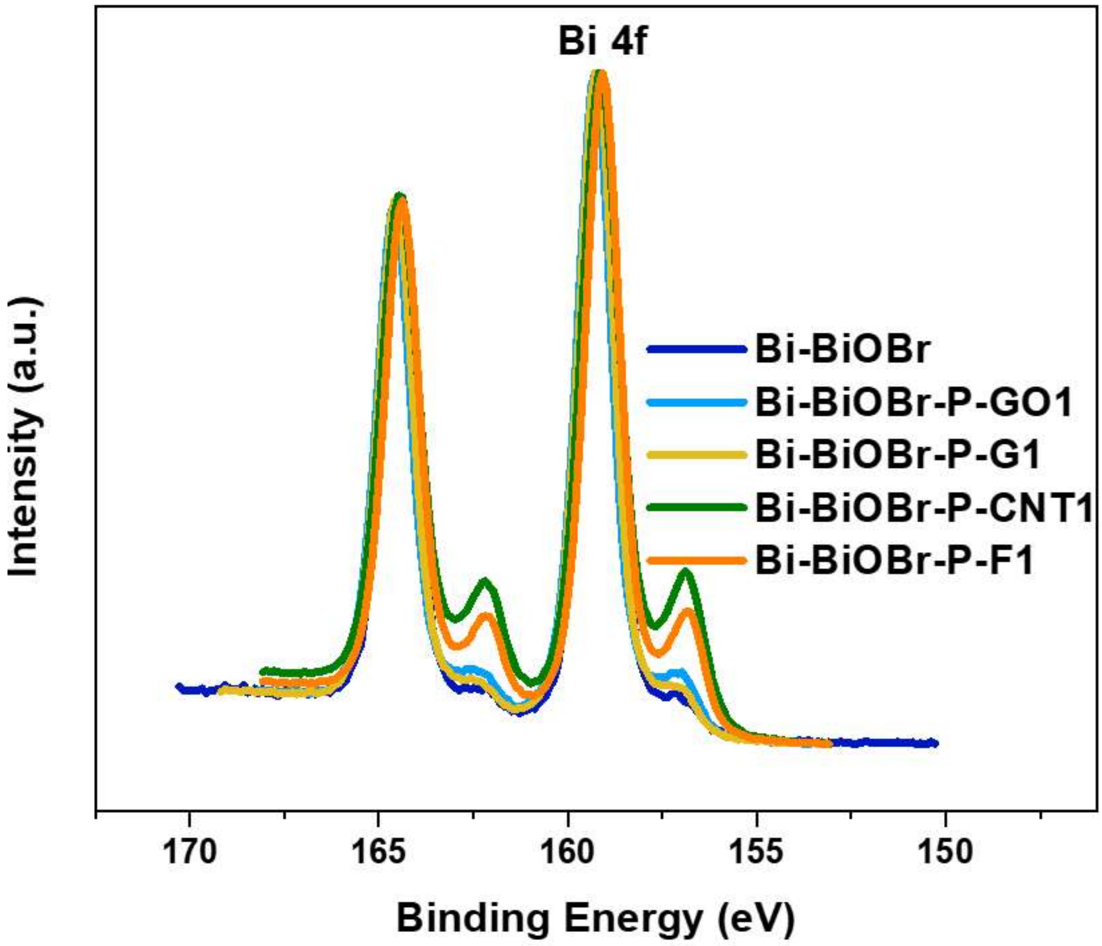

Figure S16. *Comparing the XPS Bi 4f of the Bi-BiOBr-P-GO1,Bi-BiOBr-P-G1, Bi-BiOBr-P-CNT1, and Bi-BiOBr-P-F1 composites with Bi-BiOBr.*

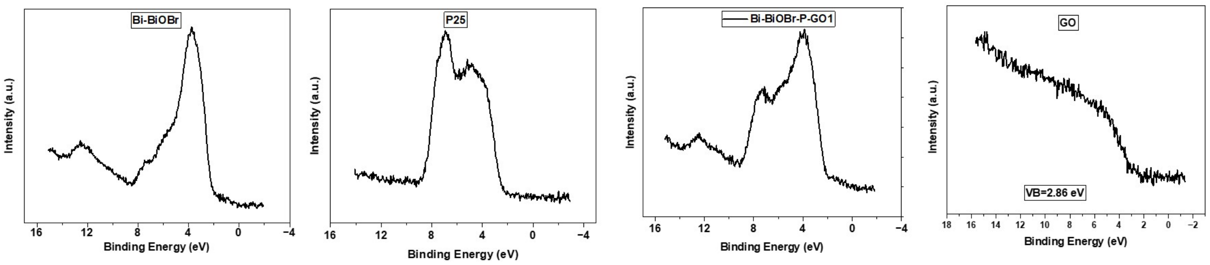

Figure S17: *Low binding energy X-ray photoelectron spectra of Bi-BiOBr, P25, Bi-BiOBr-P-GO1, and GO.*

Table S1. *A summary of the binding energies of the key environments found in Bi-BiOBr, P25, GO, G, CNT, F, Bi-BiOBr-P-GO1 (BBPGO), Bi-BiOBr-P-G1 (BBPG), Bi-BiOBr-P-CNT1 (BBPCNT), and Bi-BiOBr-P-F1 (BBPF) from X-ray photoelectron spectroscopy studies [13].*

	Binding energy (eV)									
	**Bi-BiOBr**	**P25**	**GO**	**G**	**CNT**	**F**	**BPGO**	**BPG**	**BPCNT**	**BPF**
**Br $3d_{5/2}$ Br^{1-}**	68.2	-	-	-	-	-	68.19	68.21	68.18	68.13
**Br $3d_{3/2}$ Br^{1-}**	69.3	-	-	-	-	-	69.25	69.26	69.23	69.18
**Bi $4f_{7/2}$ Bi^{3+}**	159.2	-	-	-	-	-	159.19	159.24	159.12	159.07
**Bi $4f_{5/2}$ Bi^{3+}**	164.5	-	-	-	-	-	164.51	164.56	164.43	164.38
**Bi $4f_{7/2}$ $Bi^{(0)}$**	157.2	-	-	-	-	-	157.18	157.29	156.96	156.95
**Bi $4f_{7/2}$ $Bi^{(0)}$**	162.6	-	-	-	-	-	162.48	162.62	162.26	162.25
**C 1s C=C**	-	-	284.5	284.5	284.5	284.45	284.41	284.41	284.5	284.5
**C 1s C-C**	284.8	284.8	285	284.9	284.9	284.85	285.01	285.01	285.11	285.1
**C 1s C-OH/C-O-C(C-O)**	286.4	286.1	286.7	286.4	286.4	286.25	286.41	286.31	286.41	286.5
**C 1s C=O**	288.5	-	288.1	288	288	288.45	288.2	288.21	288.01	288
**C 1s O-C=O**	-	289.2	289.1	289	289	288.9	288.91	288.91	289.01	289
**C1s π to π* Sat**	-	-	291	290.9	290.9	290.86	290.82	290.82	290.92	290.91
**Ti $2p_{3/2}$ Ti^{4+}**	-	458.9	-	-	-	-	458.73	458.8	458.78	458.78
**O 1s M-O**	530.1	530.2	-	-	-	-	530	530.03	529.97	529.96
**O 1s O-C**	531.5	531.2	532.5	531.6	531.6	532	531.6	531.34	531.56	531.52
**O 1s C=O**	533.1	-	533.5	533.1	533.1	532.7	-	-	-	-

Table S2. *The atomic percentage of the present elements in Bi-BiOBr, P25, GO, G, CNT, F, Bi-BiOBr-P-GO1 (BBPGO), Bi-BiOBr-P-G1 (BBPG), Bi-BiOBr-P-CNT1 (BBPCNT), and Bi-BiOBr-P-F1 (BBPF)measured using XPS [13].*

	**Abundance (%)**									
	**Bi-BiOBr**	**P25**	**GO**	**G**	**CNT**	**F**	**BPGO**	**BPG**	**BPCNT**	**BPF**
**Br**	6.06	-	-	-	-	-	5.05	6.2	3.44	4.36
**Bi^{3+}**	88.04	-	-	-	-	-	6.89	8.76	5.38	6.63
**Bi0**	0.46	-	-	-	-	-	1.04	1.04	1.57	1.52
**C (sp^2)**	-	-	0.83	81.38	82.1	66.07	4.14	4.5	11.69	9.92
**C (sp^3)**	-	-	83.44	16.28	16.42	33.04	2.07	2.25	2.42	4.96
**Ti**	-	47.43	-	-	-	-	35.11	35.31	31.73	31.77
**O**	5.44	52.57	15.73	2.35	1.47	0.88	45.7	41.94	43.78	40.83

PL analysis

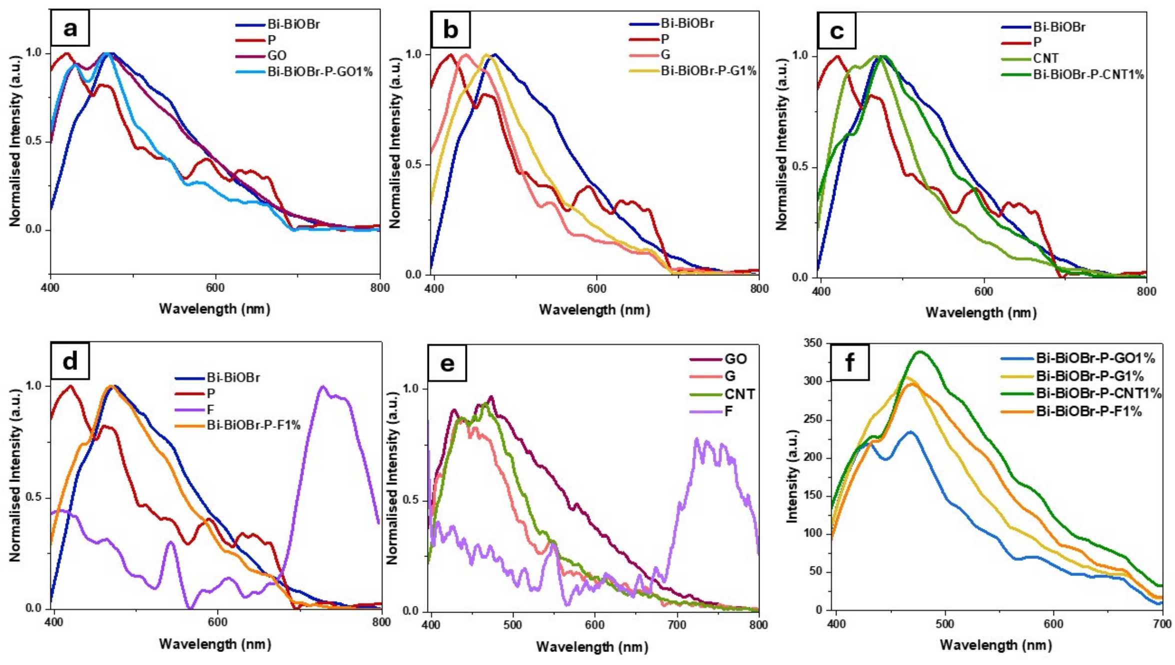

Figure S18. *Photoluminescence (PL) emission over the 390-800 nm range after excitation with 375 nm light for a) Bi-BiOBr-P-GO1, b) Bi-BiOBr-P-G1, c) Bi-BiOBr-P-CNT1, d) Bi-BiOBr-P-F1 composites compared to their parent materials, and e) carbon-based materials, and f) all composites (unnormalized).*

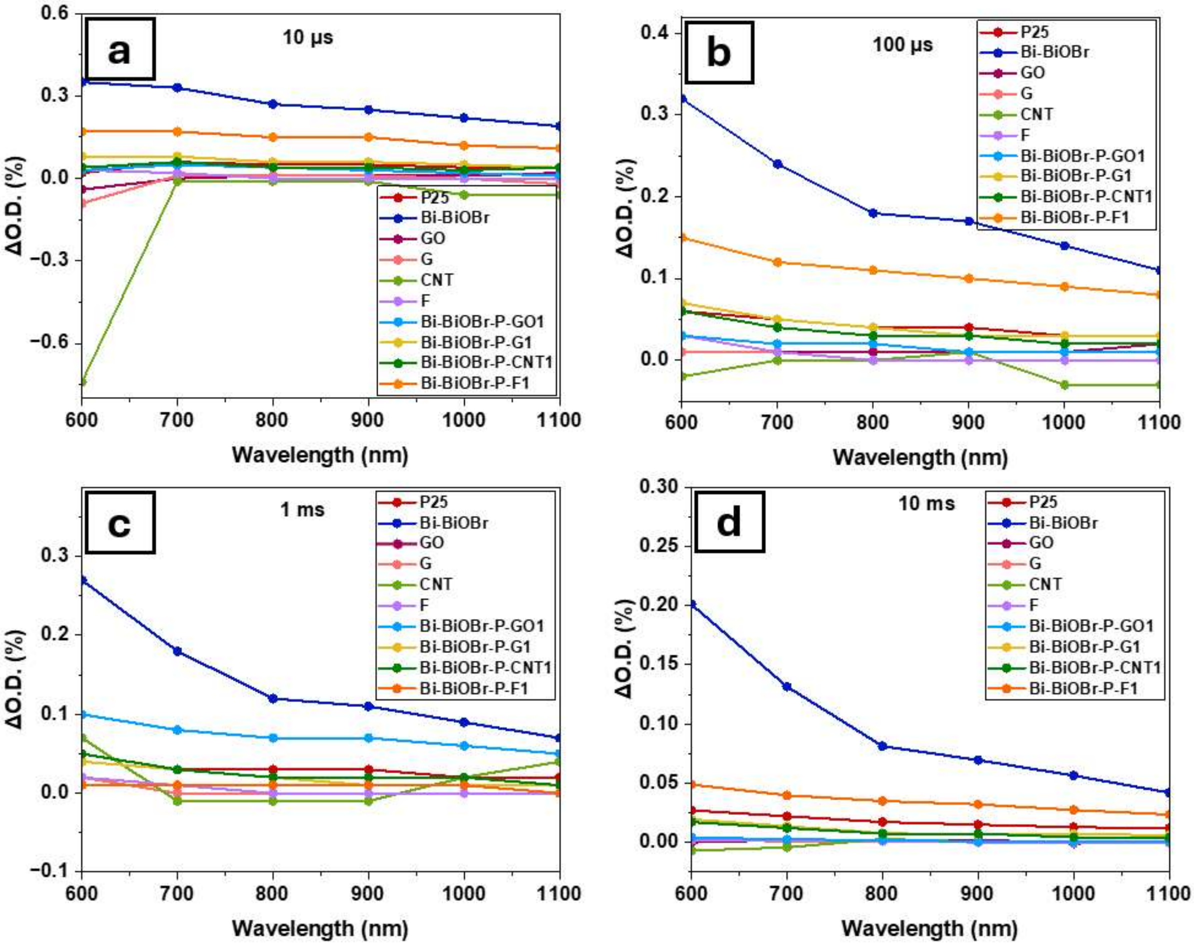

TAS analysis

Figure S19. *Transient absorption spectra of GO, G, CNT, F, Bi-BiOBr, P25, Bi-BiOBr-P-GO1, Bi-BiOBr-P-G1, Bi-BiOBr-P-CNT1, and Bi-BiOBr-P-F1 at a) 10 µs, b) 100 µs, c) 1ms, and d) 10 ms after 355 nm laser pulse excitation (6 ns pulse width, 0.67 Hz, ~220 µJ.cm^{-2} per pulse) at probe wavelengths from 600 to 1100 nm.*

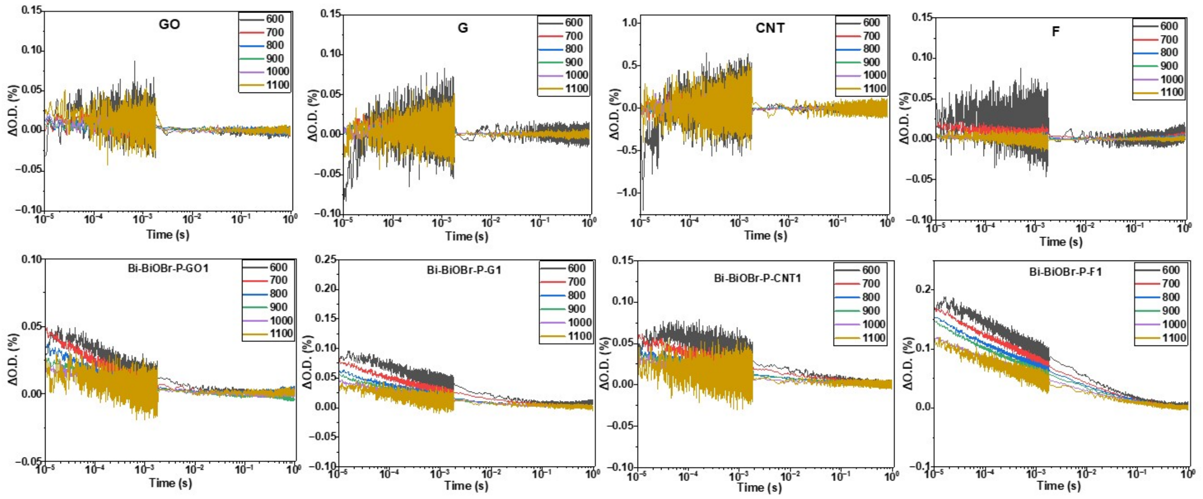

Figure S20. *Transient absorption studies of GO, G, CNT, F, P25, Bi-BiOBr, Bi-BiOBr-P-GO1, Bi-BiOBr-P-G1, Bi-BiOBr-P-CNT1, and Bi-BiOBr-P-F1. Transient absorption decay kinetics were measured from 10 μs to 1 s after excitation by a 355 nm laser pulse (6 ns pulse width, 0.67 Hz, ~220 μJ.cm-2 per pulse), with probing conducted at a wavelength of 600-1100nm [Inset: Normalized data from 10 μs].*

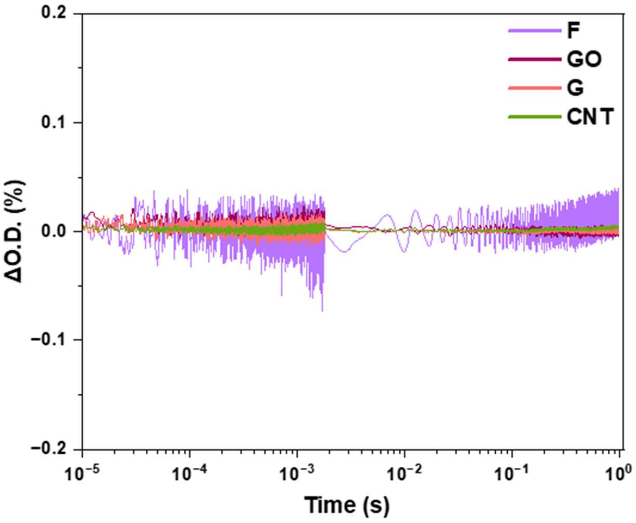

Figure S21. *Transient absorption decay kinetics for GO, G, CNT, and F samples were measured from 10 μs to 1 s after excitation with a laser (355 nm, 6ns width, ~220 μJ.cm^{-2} per pulse) and measured at a probe wavelength of 800 nm.*

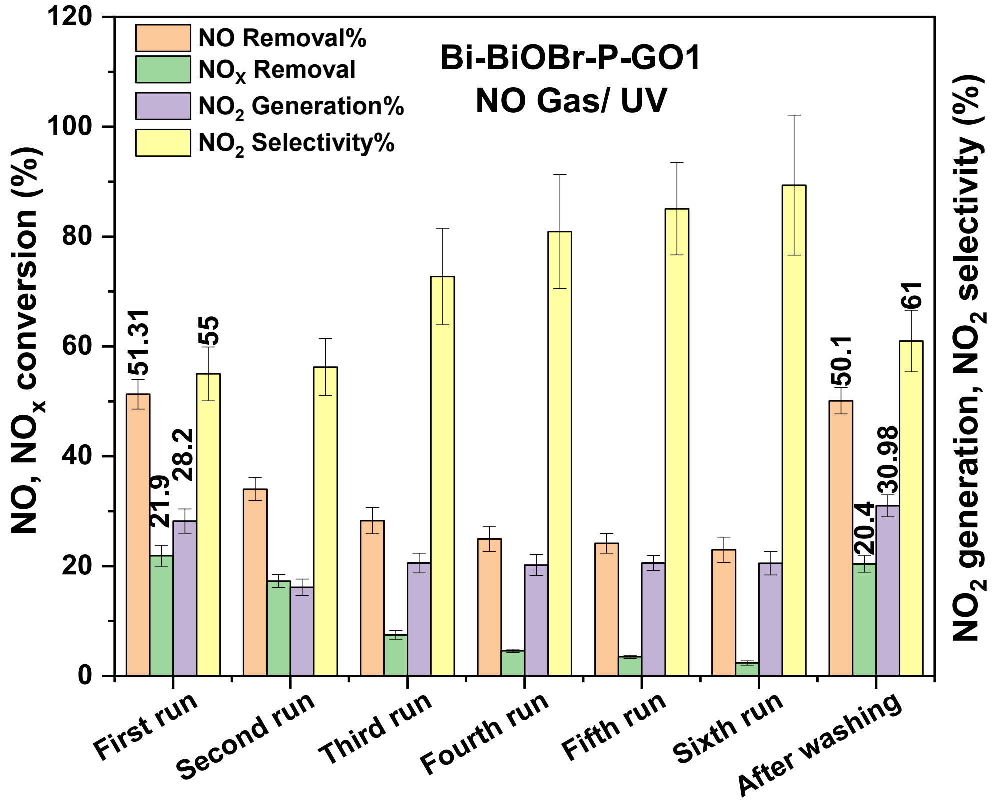

Figure S22. *Successive photocatalytic tests Bi-BiOBr-P-GO1 composite under UV light and NO gas for six successive cycles, followed by washing and drying and another photocatalytic test.*

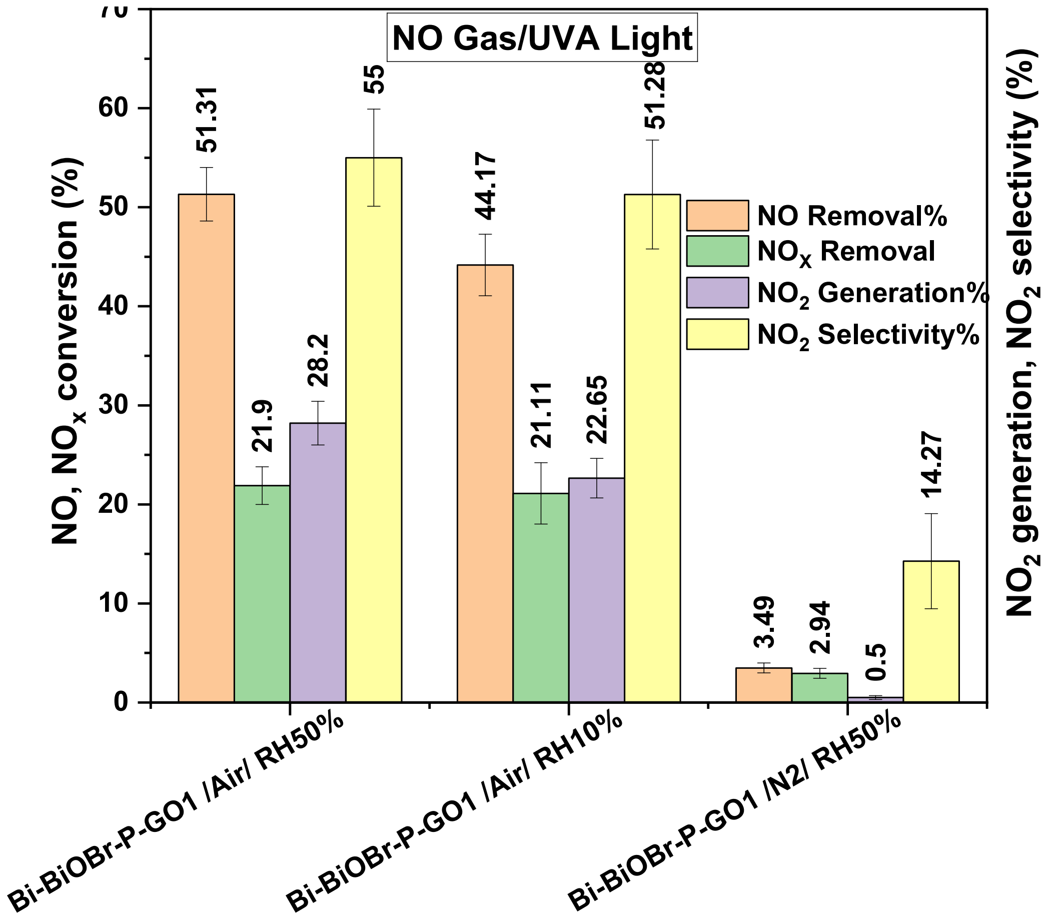

Figure S23. *Photocatalytic tests on the Bi-BiOBr-P-GO1 composite under UV light and NO gas, conducted in the presence of air and 50% relative humidity, in the presence of air and 10% relative humidity, and in the absence of air with* N_2 *and 50% relative humidity.*

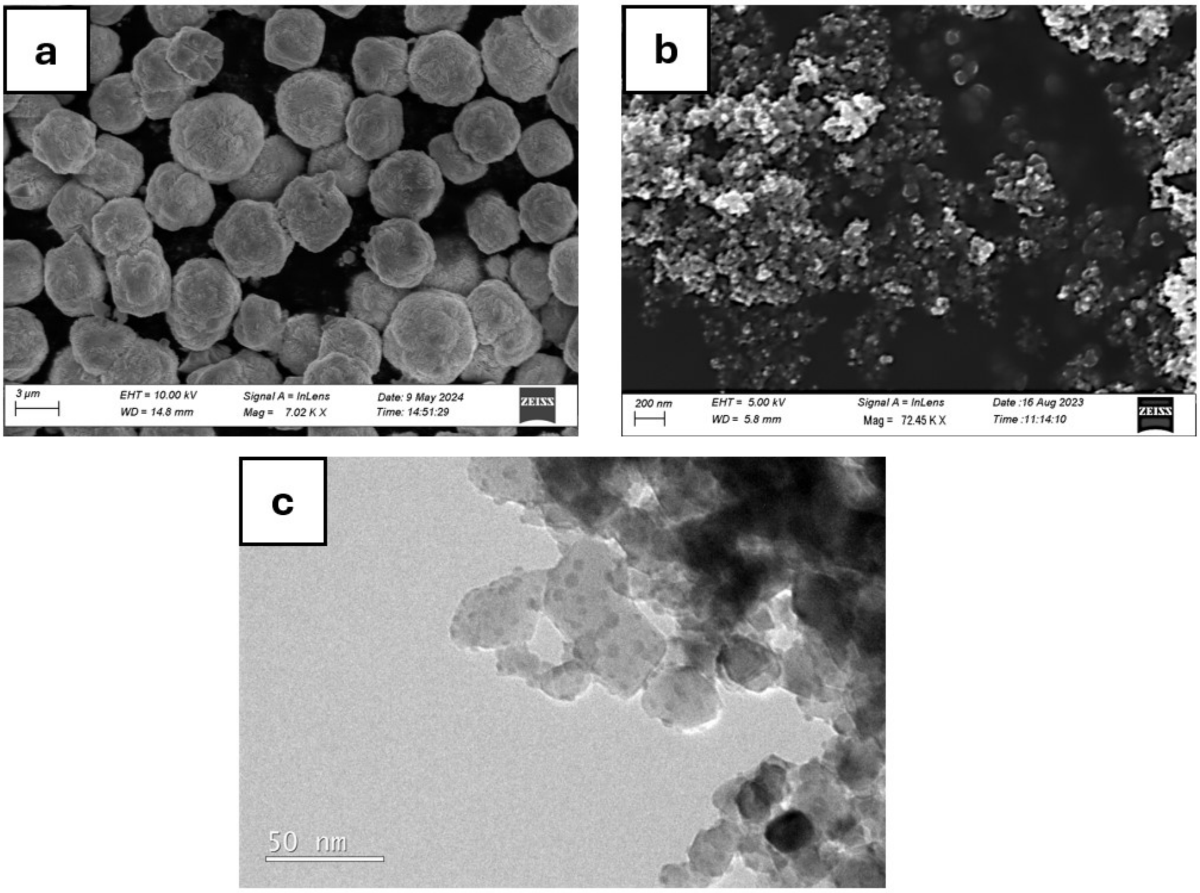

Figure S24. *SEM images of a)Bi-BiOBr and b) P25, and TEM image of c)Bi-BiOBr-P.*
